# Machine Learning Across Heterogeneous Biomedical Data: Representation, Integration, and Deployable Systems

**DOI:** 10.3390/bioengineering13060683

**Published:** 2026-06-12

**Authors:** Alin Adrian Alecu

**Affiliations:** Faculty of Engineering in Foreign Languages (FILS), National University of Science and Technology Politehnica Bucharest, Splaiul Independentei 313, 060042 Bucharest, Romania; alin_adrian.alecu@upb.ro

**Keywords:** multimodal learning, biomedical data integration, biomedical machine learning, deployment-aware machine learning, longitudinal modeling

## Abstract

Biomedical prediction increasingly requires machine learning methods capable of integrating heterogeneous data spanning molecular, physiological, clinical, behavioral, and environmental representations. Yet progress in this area has often been framed primarily in terms of predictive algorithms, with less attention placed on how biomedical data are represented, integrated, and translated into deployable systems. This review synthesizes machine learning for heterogeneous and multimodal biomedical data settings through three complementary perspectives. First, we organize the literature into four recurring prediction regimes—structured biomedical prediction, high-dimensional biomedical signals, multimodal learning, and temporal or longitudinal modeling—and review representative datasets and model families associated with each. Second, we analyze recurrent pipeline architecture motifs, highlighting trends in handcrafted feature pipelines, learned representations, staged predictive systems, and robustness-aware architectures. Third, we examine biomedical machine learning as a constrained systems design problem shaped by partial observability, alignment challenges, robustness, and deployment requirements. Across regimes, a central theme emerges: effective biomedical machine learning depends not only on model choice, but also on principled design of representations, information flow, modularity, and deployability. The reviewed approaches emphasize multimodal integration and predictive learning rather than explicit mechanistic scale-coupling models.

## 1. Introduction

Modern biomedical research increasingly depends on the integration of heterogeneous data representations spanning molecular measurements, physiological signals, clinical records, behavioral observations, and environmental context [1,2]. These data sources differ substantially in modality, dimensionality, temporal structure, resolution, reliability, and accessibility, creating major computational challenges for predictive modeling and translational deployment.

Recent advances in biomedical data acquisition have made such integration increasingly feasible [1]. High-dimensional molecular measurements, medical imaging, physiological waveforms, behavioral and survey data, digital phenotyping, wearable sensing, and large longitudinal cohorts now provide unprecedented opportunities to study health across heterogeneous biomedical domains [1,3]. At the same time, this diversity of data has created new computational demands, particularly for methods capable of integrating partially observed, temporally irregular, multimodal, and longitudinal information under realistic deployment constraints.

Concepts such as allostatic load have helped formalize relationships between psychosocial processes, physiological adaptation, and measurable biological dysregulation across time [4,5,6]. These settings illustrate why biomedical prediction often cannot be reduced to isolated modalities or single physiological systems alone. Psychosocial exposures, behavioral dynamics, neuroendocrine regulation, metabolic function, and long-term physiological adaptation interact over time, producing distributed patterns of biological and clinical variation represented across heterogeneous biomedical measurements. From a machine-learning perspective, these challenges motivate predictive systems capable of integrating multimodal and longitudinal data while remaining sufficiently robust for deployment in real-world clinical and population-health settings.

Machine learning (ML) has emerged as a central framework for addressing these challenges. Classical statistical learning, tree-based methods, deep neural networks, as well as more recent multimodal fusion and temporal architectures, have been increasingly applied to heterogeneous biomedical data representations [2,7,8]. Yet these methods are often studied in separate studies organized by modality or task, making it difficult to understand how they relate as components of broader biomedical machine-learning systems.

A central premise of this review is that effective biomedical machine learning is not defined simply by the use of multiple modalities or sophisticated predictive models, but by the structured transformations through which heterogeneous measurements are represented, integrated, and translated into predictive systems [2,8]. Although related to broader multiscale modeling efforts in systems biology and computational physiology, the present review focuses primarily on predictive machine-learning systems operating across heterogeneous biomedical data representations, emphasizing representation learning, multimodal integration, longitudinal modeling, robustness, and deployability rather than explicit mechanistic scale-coupling frameworks. From this perspective, the focus shifts from model choice alone toward questions of representation, information flow, modularity, predictive inference, and deployability.

A second motivating issue is the persistent gap between model development and real-world deployment. Many high-performing models are trained on rich multimodal or longitudinal datasets that differ substantially from the limited, noisy, and often non-invasive data available in practical screening or monitoring settings. This mismatch creates a recurring tension between model expressivity and deployability, amplified by challenges involving missing modalities, distribution shift, interpretability, and operational constraints [9,10].

In this context, this review examines machine learning across heterogeneous and multimodal biomedical data settings through three complementary perspectives. The proposed prediction regimes and systems-design perspectives are intended as organizational and interpretive constructs for cross-domain synthesis rather than as rigid or exhaustive categorical taxonomies. First, we organize the literature around four recurring prediction regimes—structured biomedical prediction, high-dimensional biomedical signals, multimodal learning, and temporal or longitudinal modeling—and analyze representative datasets and model families associated with each. Second, we examine biomedical machine-learning systems from a systems-design perspective, emphasizing recurrent architectural motifs involving representation learning, fusion, staged prediction, and deployment-aware pipelines. Third, we discuss the broader methodological and translational challenges that arise when moving from predictive models toward robust and deployable biomedical machine-learning systems.

Throughout, particular emphasis is placed on a recurring theme: progress in biomedical machine learning depends not only on improving algorithms, but on reconciling representation, robustness, and deployment under real-world constraints. Approaches such as adaptive multimodal modeling [11], knowledge transfer [12], and teacher–student learning [13] are discussed in this broader context not simply as algorithmic techniques, but as emerging components of deployable biomedical machine-learning systems.

By organizing recurring relationships among data regimes, model families, architectural patterns, and deployment constraints across heterogeneous biomedical domains, this review aims to provide an integrative perspective on representation, integration, and deployable biomedical machine-learning systems.

## 2. Multilevel Data Representations in Health Modeling

A defining feature of health-related data is that relevant information is distributed across multiple biological, physiological, behavioral, and environmental levels of organization, spanning molecular measurements, physiological signals, clinical records, behavioral assessments, digital sensing streams, and environmental context. These modalities differ not only in content but also in dimensionality, temporal structure, observability, and accessibility, making biomedical modeling as much a problem of representation and data integration as of prediction.

A central distinction throughout this review is between the available multilevel data space of potentially informative modalities and the deployable subset that can realistically be acquired in practice. While modern datasets may support rich multimodal representations, deployed systems often operate under constraints on modality availability, cost, and invasiveness. This gap between informational richness and practical accessibility has major implications for model design and deployment.

### 2.1. Biomedical Data Modalities Across Representation Levels

Biomedical data used in predictive machine-learning systems span heterogeneous modality classes associated with different forms of biological measurement, clinical abstraction, behavioral observation, and environmental context. These representation levels should not be interpreted as formal mechanistic scales linked through explicit dynamical coupling, but rather as distinct categories of biomedical information that differ in modality, temporal structure, dimensionality, accessibility, and deployment characteristics. Table 1 summarizes representative categories, their typical data structures, temporal characteristics, and relative accessibility.

These modalities can be organized along a continuum of biological proximity, observational context, temporal resolution, and deployment accessibility, ranging from molecular and imaging measurements to behavioral and environmental representations. Lower-level modalities often provide biologically proximal information but tend to be costly, invasive, high-dimensional, and temporally sparse. In contrast, behavioral and contextual modalities are often more accessible and scalable, but may be noisier or provide indirect views of the system. Physiological and clinical modalities frequently occupy an intermediate position, combining structured variables with temporal dynamics and supporting both mechanistic and predictive modeling. Within predictive biomedical machine-learning systems, these modalities are therefore best understood as complementary representations of health-related variation that impose different modeling assumptions, information constraints, and deployment trade-offs rather than as explicit mechanistic scales connected through formal dynamical scale-coupling frameworks.

### 2.2. Representative Datasets Across Modalities

The development of machine-learning systems operating across heterogeneous biomedical data levels has been closely linked to the availability of datasets spanning different modality classes. However, data availability is highly uneven across scales, and predictive modeling is often shaped as much by available data resources as by methodological considerations.

At the biological level, resources such as TCGA [14] and GTEx [15] provide large-scale molecular measurements, while imaging cohorts such as UK Biobank imaging [16], ADNI [17], and digital pathology datasets such as CAMELYON [18] support high-dimensional spatial modeling and representation learning.

Physiological and clinical data are represented in resources such as MIMIC-III/IV [19,20], eICU [21], and large electronic health record repositories, often combining irregular longitudinal measurements with structured and unstructured clinical information.

Longitudinal cohort studies, including MIDUS [22,23], HRS [24], ELSA [25], and NHANES [26], provide integrated behavioral, demographic, clinical, and biomarker data for studying health trajectories over time, though often with sparse temporal resolution and incomplete modality coverage.

Digital phenotyping datasets such as StudentLife [27], mPower [28], and RADAR-CNS [29] have introduced continuous behavioral sensing data derived from smartphones and wearable devices, while contextual information may be linked through external environmental data sources such as OpenAQ [30] and NOAA [31].

Despite this breadth, few datasets provide comprehensive coverage across all modalities. Most are concentrated within specific levels of the biological and behavioral hierarchy, and multimodal combinations are often constrained by study design, acquisition burden, and missingness. Consequently, models are frequently developed on subsets of the full modality space and may depend on information unavailable in deployment settings.

This imbalance in data availability highlights a central challenge in biomedical machine learning: rich multimodal datasets may enable expressive models, while practical applications often operate on a smaller subset of accessible modalities. This discrepancy motivates modeling strategies that explicitly account for modality availability, as discussed further in this paper.

### 2.3. Structural Properties of Multilevel Biomedical Data

Biomedical data spanning multiple organizational levels are inherently heterogeneous, not only in biological interpretation but also in statistical structure, temporal resolution, and availability. These properties directly influence how data can be represented, integrated, and used in machine learning models.

A fundamental distinction arises from differences in data structure and dimensionality. Lower-level modalities, such as omics and imaging, are typically high-dimensional and often require representation learning to extract useful features. In contrast, higher-level modalities—including clinical variables, anthropometrics, and survey measures—are structured and often usable directly as model inputs. Digital sensing data occupy an intermediate position, frequently consisting of high-frequency signals requiring aggregation or sequence modeling.

Temporal resolution also varies substantially across modalities. Molecular and imaging data are often static or episodic, clinical and behavioral data may form sparse irregular longitudinal trajectories, and digital sensing or environmental data may generate dense continuous streams [27,29,31]. As a result, multimodal biomedical modeling often involves integrating asynchronous data sources with fundamentally different temporal properties.

Data quality and uncertainty likewise differ across modalities. Clinical and biomarker measurements may be relatively reliable but subject to measurement variability, behavioral data may be influenced by reporting biases [32], and digital sensing data may introduce variability due to device heterogeneity and user behavior [3]. Environmental data may introduce further uncertainty through spatial or temporal proxy linkage.

A defining feature of heterogeneous biomedical data is the prevalence of missingness and partial observability. Missing data are often structured rather than random and may arise from study design, acquisition costs, or user behavior. Importantly, missingness frequently occurs at the modality level, with entire data types absent for subsets of individuals.

These conditions can be formalized by considering the set of available modalities M and the subset S⊆M observed for a given individual or deployment setting. In practice, both the composition of *S* and the quality of observations within each modality may vary substantially across contexts. As a result, biomedical machine-learning systems must operate under conditions of heterogeneous, incomplete, and partially aligned data.

### 2.4. Challenges for Cross-Level Integration and Modeling Constraints

The properties of heterogeneous biomedical data translate directly into constraints on machine learning models. In particular, the observed modality set S⊆M is rarely fixed across samples or deployment settings and may differ substantially from the full modality set available during model development. This mismatch creates a fundamental challenge: models trained on rich multimodal data may implicitly rely on information unavailable at inference time.

Beyond modality availability, integration itself is complicated by differences in scale, semantics, sampling frequency, and noise characteristics across data sources. Missing modalities, asynchronous measurements, and partial observability make cross-level relationships difficult to represent, while temporal irregularity and modality imbalance further complicate learning. In many applications, the central challenge lies less in prediction from any single modality than in reconciling incomplete and heterogeneous views of the same biological system.

Taken together, these constraints motivate the regime-based organization adopted in the following section, where machine learning methods are examined across structured, high-dimensional, multimodal, and temporal prediction settings. More broadly, the present review focuses on predictive systems operating under heterogeneous data and deployment constraints rather than on explicit mechanistic scale-coupling models.

## 3. Machine Learning Approaches

Machine learning in the biological, biomedical, and behavioral sciences is not defined by a single dominant modeling paradigm, but by a set of approaches that emerge in response to the heterogeneous biomedical data spanning multiple biological, clinical, behavioral, and environmental levels of representation. As described in the previous section, health-related data span multiple levels of organization, differ substantially in dimensionality and temporal structure, and are often incomplete or only partially observable. These characteristics impose distinct constraints on model design, leading to different classes of methods suited to different data regimes.

Accordingly, this section organizes machine learning approaches through a data- and constraint-driven framework, emphasizing how modeling paradigms relate to the statistical structure of heterogeneous biomedical data and to the requirements of deployment. In this framing, machine learning models are not treated as interchangeable tools, but as responses to specific combinations of modality, data availability, and prediction task.

**Figure 1:**
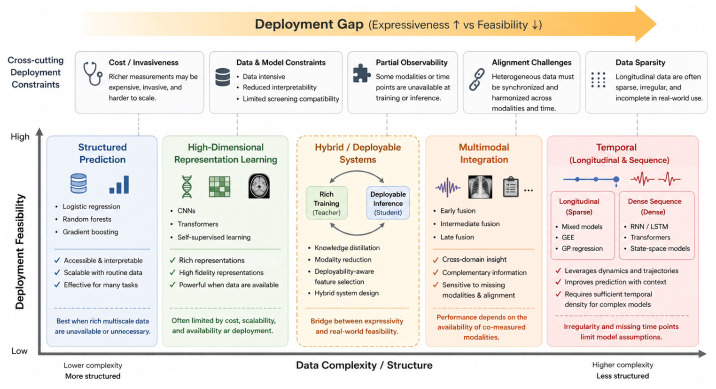
Conceptual view of machine-learning regimes across heterogeneous biomedical data settings along the expressivity–deployability axis. Structured, high-dimensional, multimodal, and temporal approaches vary in data complexity, alignment, and robustness to partial observability, while cross-cutting constraints (cost, sparsity, and heterogeneity) shape real-world feasibility. Hybrid systems bridge this gap by combining rich training-time models with deployable inference-time representations.

Figure 1 illustrates the relationship between model expressiveness and deployment feasibility across major machine learning regimes in biomedical prediction systems operating across heterogeneous data modalities. Rather than a strict progression, structured, high-dimensional, multimodal, and temporal approaches represent distinct modeling settings that differ in data requirements, scalability, and robustness to partial observability. While richer representations can capture more complex biological structure, they also introduce constraints related to data availability, alignment, and deployment, giving rise to a deployment gap. Hybrid and deployable system designs can help bridge this gap by combining expressive training-time models with constrained inference-time inputs.

The following subsections examine these regimes in turn, highlighting their characteristic modeling approaches, empirical usage patterns, and the constraints that shape their role in deployable biomedical systems.

### 3.1. Prediction Regimes Across Heterogeneous Biomedical Data

Differences in data structure, dimensionality, temporal resolution, and modality availability give rise to a small number of recurring prediction regimes that reflect not only biological levels of measurement, but also the statistical and operational constraints under which data are collected and used.

Table 2 provides an illustrative mapping between representative datasets and these regimes, reflecting their predominant use in the literature and how different combinations of modalities and data characteristics give rise to distinct modeling settings.

A large proportion of work in epidemiology and population health operates in the structured biomedical prediction regime, where inputs consist of low- to moderate-dimensional tabular variables derived from clinical records, surveys, or cohort studies. These settings often combine heterogeneous demographic, clinical, behavioral, and environmental variables, leading modeling approaches to emphasize robustness to missingness, interpretability, and mixed feature types [33,34].

In contrast, high-dimensional signal modeling arises from modalities such as imaging and omics, which occupy the biologically proximal representation levels. These data are characterized by large input dimensionality and complex spatial or molecular structure, requiring representation learning to extract meaningful features. As a result, model design in this regime is driven by the need to learn hierarchical representations directly from raw data [35,36].

A third regime involves temporal and longitudinal prediction, which emerges when data exhibit irregular trajectories, continuous streams, or evolving system states. In these settings, the challenge lies not merely in incorporating time, but in modeling temporal dependencies under asynchronous and uneven sampling [2].

Finally, multimodal prediction arises when information from multiple modalities is integrated, such as combining structured variables with high-dimensional signals or incorporating behavioral and environmental data alongside clinical measurements. These settings introduce additional challenges related to modality alignment, heterogeneous feature spaces, and incomplete modality availability at inference time [8,37].

These regimes are not mutually exclusive and often co-occur within the same application. Nevertheless, each reflects a distinct set of constraints inherited from the underlying data and motivates different families of machine learning methods examined in the following subsections.

### 3.2. Data Collection and Computational Literature-Mining Workflow

**Figure 2:**
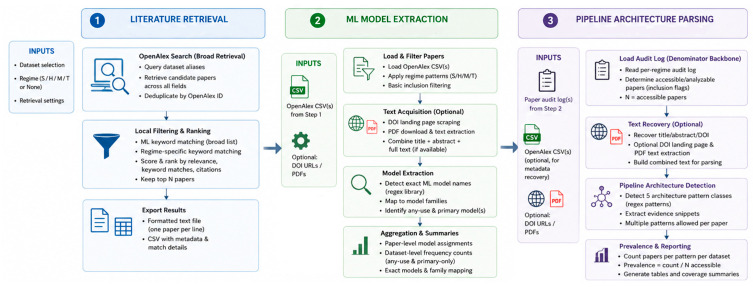
Computational literature-mining workflow used for dataset retrieval, regime filtering, model extraction, dominant-model assignment, validation auditing, and prevalence-oriented model-family analysis across the four prediction regimes.

To characterize machine learning usage across heterogeneous biomedical datasets spanning multiple representation levels, we constructed dataset-specific corpora of machine learning studies using the OpenAlex scholarly database [38]. The overall computational workflow is summarized in Figure 2. The study should be interpreted as a computational literature-mapping review rather than a formal systematic review or meta-analysis.

#### 3.2.1. Literature Retrieval and Corpus Construction

OpenAlex retrievals were performed on 19 April 2026 using the OpenAlex API and dataset-specific query configurations. For each dataset, multiple query variants were defined based on common aliases and naming conventions (e.g., “MIMIC-III”, “MIMIC-IV”, “Medical Information Mart for Intensive Care”). Candidate records were retrieved through broad metadata-based searches over titles and abstracts. The retrieval strategy intentionally favored sensitivity over specificity in order to capture heterogeneous terminology across biomedical machine learning studies. Retrieved records from different aliases were subsequently deduplicated using OpenAlex identifiers before downstream analysis.

To focus the corpora on machine learning applications, retrieved papers were filtered using an extensive machine learning keyword library containing both general and model-specific terminology, including terms such as “machine learning”, “deep learning”, “random forest”, “support vector machine”, “survival analysis”, and “neural network”. In addition, regime-specific keyword and pattern libraries were used to bias retrieval toward the targeted prediction setting. Structured-regime filtering included terms related to cohorts, surveys, electronic health records, and tabular prediction; high-dimensional filtering emphasized imaging and omics terminology; multimodal filtering targeted data fusion and heterogeneous modalities; and temporal filtering emphasized longitudinal and time-series concepts. Representative examples of these pattern libraries are provided in the Supplementary Materials together with the complete rule sets.

For each dataset–regime pair, candidate papers were ranked using a composite heuristic combining dataset mention, machine learning keyword matches, regime-specific pattern matches, OpenAlex relevance scores, and citation counts. In most cases, the highest-ranked ∼350 papers for each dataset–regime pair were retained for downstream analysis. However, for a small number of sparse dataset–regime combinations yielding limited numbers of relevant papers after downstream filtering, the initial OpenAlex retrieval depth was increased (up to approximately 800 candidate records) to improve corpus coverage and maintain sufficient analyzable samples. The resulting corpora were subsequently processed using a multi-stage rule-based text analysis pipeline.

Several sparse dataset–regime combinations remained limited in size even after retrieval expansion. These low-yield combinations were retained because they may themselves reflect meaningful properties of the underlying literature landscape rather than only retrieval limitations. In particular, sparse retrieval may indicate that some assumed associations between representative datasets and prediction regimes are comparatively weak within the published machine-learning literature. This is especially relevant because Table 2 was intended as a conceptual mapping of representative datasets to prediction regimes rather than an empirically validated classification. We additionally observed that the multimodal regime generally produced smaller retained corpora than the other regimes, which may partly reflect the comparatively heterogeneous terminology used across multimodal machine-learning studies. Accordingly, percentages derived from sparse dataset–regime combinations should be interpreted cautiously and primarily as descriptive indicators rather than statistically stable prevalence estimates.

#### 3.2.2. Rule-Based Model and Regime Extraction

Each paper was represented by its title and abstract, optionally augmented with additional text retrieved from DOI landing pages or accessible PDF content when available. A second-stage regime relevance filter was then applied using curated regular-expression pattern libraries designed to identify regime-specific methodological characteristics such as longitudinal trajectories, multimodal integration, imaging pipelines, omics representations, or sequential prediction structures. Papers were retained for further analysis only if they satisfied the target regime criteria and contained at least one identifiable machine learning model.

Machine learning models were extracted using predefined pattern-based rules that mapped textual mentions (e.g., “random forest”, “XGBoost”, “LSTM”, “Cox regression”) to standardized exact-model identifiers. Extracted models were subsequently grouped into broader model families using a locked taxonomy consisting of deep learning, tree-based ensemble methods, linear and generalized linear models, kernel or instance-based methods, probabilistic or statistical models, survival models, ensemble or stacking methods, and other classical machine learning approaches. Exact-model mappings and representative regex libraries are provided in the Supplementary Materials. This normalization procedure enabled aggregation across heterogeneous naming conventions and reporting styles used in the literature.

Two complementary analyses were performed. In the first analysis, each paper was assigned a single dominant model and dominant model family intended to represent the primary methodological approach driving the study. Dominant-model assignment relied on heuristic contextual cues applied to textual model mentions, including expressions such as “we propose”, “we trained”, “our model”, “based on”, and related formulations suggesting methodological centrality. Papers for which a unique dominant model could not be identified were excluded from dominant-model-family analyses. In the second analysis, all identifiable model families mentioned within a paper were retained, allowing each study to contribute to multiple model families simultaneously. These prevalence-oriented model-family analyses capture broader methodological usage patterns, including benchmarking studies, auxiliary feature extractors, and hybrid pipelines.

#### 3.2.3. Validation of the Computational Literature-Mining Pipeline

Because the quantitative summaries presented in subsequent sections were derived from a heuristic rule-based literature-mining framework, an additional manual audit procedure was performed to assess extraction reliability and dominant-model assignment consistency. The validation focused on three components of the computational pipeline: (i) regime-relevance filtering, (ii) prevalence-oriented model-family extraction, and (iii) dominant-model and dominant-model-family assignment. The audit was intended to evaluate the consistency of the extraction framework itself rather than to validate downstream biological or clinical conclusions.

For each prediction regime (structured, high-dimensional, multimodal, and temporal), a random audit subset of 50 papers was sampled from the intermediate audit logs after relevance filtering. Eligible papers were sampled from studies retained within the final regime-specific analysis corpora after computational relevance filtering. The audit therefore evaluated the reliability of the downstream model-extraction and dominant-family assignment procedures on the papers contributing to the quantitative summaries reported throughout the manuscript. Accessible PDF files were downloaded when available; otherwise, title and abstract text retrieved through OpenAlex metadata were used for manual inspection. Each audited paper was manually reviewed using the exact-model and model-family taxonomy described above. Manual annotations included (i) the dominant model family, (ii) the dominant exact model, (iii) ambiguity flags indicating cases without a uniquely identifiable dominant model, and (iv) all identifiable model families and exact models present within the paper.

Validation metrics were computed separately for dominant-model-family assignment and prevalence-oriented model-family extraction. Dominant-model-family validation was treated as a single-label classification problem because each paper contributed at most one dominant model family. In contrast, prevalence-oriented model-family extraction was evaluated as a multi-label extraction problem because individual papers could contain multiple model families simultaneously. $A_{\mathrm{dom}}$ therefore evaluates dominant-family accuracy, whereas $P_{\mathrm{fam}}$, $R_{\mathrm{fam}}$, and $F1_{\mathrm{fam}}$ evaluate the precision, recall, and F1 score of prevalence-oriented model-family extraction, respectively. The resulting validation metrics across the four prediction regimes are summarized in Table 3.

The manual audit demonstrated reasonable agreement between manually reviewed annotations and automated extraction outputs, particularly for prevalence-oriented model-family extraction. Validation performance was generally strongest in the structured regime, likely because structured biomedical prediction studies more frequently employ comparatively standardized methodological descriptions centered on tabular machine-learning pipelines. Across regimes, prevalence-oriented family extraction achieved consistently high recall, indicating that the pipeline recovered most manually identified model-family assignments within the audited subsets. Dominant-family assignment proved more challenging in high-dimensional studies, where imaging and omics papers often contain hybrid deep-learning architectures, transfer-learning stages, auxiliary representation modules, and multistage computational pipelines that complicate identification of a single dominant model family. Because the validation was performed on relatively small randomly sampled audit subsets from substantially larger corpora, these results should be interpreted as approximate indicators of pipeline behavior rather than definitive estimates of extraction performance across the entire literature.

To further evaluate the robustness of the literature-mining framework to taxonomy design choices, an additional sensitivity analysis was performed using multiple coarser model-family aggregation scenarios. These analyses applied alternative family-collapsing schemes simultaneously to both automated predictions and manually annotated labels, thereby testing whether the overall conclusions depended strongly on fine-grained distinctions among adjacent model families. The evaluated scenarios included (i) collapsing multiple classical machine-learning families into broader aggregate categories, (ii) merging ensemble-oriented families, (iii) merging survival and linear/statistical families, and (iv) collapsing all non-deep-learning families into a single category.

As summarized in Table 4, prevalence-oriented extraction performance remained comparatively stable across the evaluated taxonomy-collapse scenarios, particularly in the structured and temporal regimes. The largest sensitivity effects were observed in the high-dimensional regime, where imaging and omics studies more frequently contained overlapping hybrid architectures and less sharply separated methodological categories. Nevertheless, even under comparatively aggressive family-collapsing schemes, the resulting changes in prevalence-oriented extraction performance remained moderate, suggesting that the broad methodological patterns reported throughout the manuscript were not strongly dependent on a single fine-grained taxonomy definition.

The validation process additionally revealed that ambiguity in dominant-model-family assignment was itself a meaningful characteristic of the literature landscape rather than solely a parsing failure. In many studies, methodological contributions were distributed across hybrid pipelines, ensemble architectures, feature-engineering stages, or comparative benchmarking designs without a single clearly dominant predictive model. These observations motivated the complementary use of both dominant-model-family summaries and prevalence-oriented model-family analyses throughout the manuscript. Formal inter-rater agreement statistics were not computed because the manual audit was performed by a single annotator operating under a predefined taxonomy and standardized annotation protocol.

#### 3.2.4. Aggregation, Pipeline Patterns, and Reproducibility

Model-family statistics were aggregated separately for each dataset and prediction regime. The primary quantitative outputs included prevalence-oriented model-family percentages, dominant-model-family percentages, exact-model counts, auxiliary architectural motif statistics, and corpus coverage summaries. Dominant-model-family analyses were expressed as percentages of papers assigned to each primary model family, while prevalence-oriented model-family analyses represented the percentage of papers containing at least one model belonging to a given family. Because individual papers could contain multiple model families simultaneously, prevalence-oriented percentages are not constrained to sum to 100%.

In addition to model-family extraction, supplementary rule-based parsing procedures were used to identify higher-level architectural and pipeline motifs, including handcrafted feature pipelines, learned representation pipelines, fusion-centric architectures, sequential predictive pipelines, and transfer-aware or robustness-oriented workflows. These motif categories were intended primarily as descriptive architectural indicators rather than formally validated ontological classes. Motif identification relied on heuristic pattern matching applied to titles, abstracts, DOI-accessible textual content, and accessible PDF text when available. Because many studies contained overlapping architectural characteristics, heterogeneous reporting conventions, or partially described pipelines, the resulting motif prevalence statistics should be interpreted cautiously as exploratory summaries of recurrent design tendencies rather than precise quantitative prevalence estimates. Accordingly, the auxiliary motif analysis was used primarily to contextualize broad architectural trends observed across prediction regimes rather than to support strong inferential claims regarding dominance of specific pipeline paradigms.

Pipeline-pattern prevalence statistics were computed analogously to prevalence-oriented model-family analyses using the proportion of accessible papers containing at least one identifiable architectural motif. Because papers could simultaneously contain multiple architectural characteristics, motif prevalence percentages were likewise not constrained to sum to 100%. The auxiliary motif analysis was therefore intended to complement the dominant-model-family and prevalence-oriented model-family analyses by providing a higher-level descriptive view of recurring computational design structures observed across biomedical machine-learning studies.

To improve transparency and reproducibility, the computational pipeline generated detailed audit logs, intermediate count tables, model-family mapping files, validation audit summaries, coverage statistics, and dataset-specific parsing outputs. The complete computational literature-mining framework, including source code, keyword libraries, regime-specific pattern sets, model-family mappings, dominant-model assignment heuristics, validation audit procedures, representative parsing outputs, and the manually annotated audit subsets used for validation, has been released in a public GitHub repository (https://github.com/AAAlecu/biomedical-ml-literature-mapping, accessed on 24 May 2026) and is additionally documented in the Supplementary Materials. The released audit resources include the retained regime-specific validation subsets together with their corresponding manual annotations and associated paper-retrieval metadata.

### 3.3. Structured Biomedical Prediction

Structured, tabular data remain a central paradigm in biomedical and population-level machine learning. Large cohort studies such as MIDUS, HRS, ELSA, UK Biobank, and NHANES provide rich collections of demographic, clinical, behavioral, and psychosocial variables. Similarly, electronic health record (EHR) systems such as MIMIC and eICU integrate diagnostic codes, laboratory measurements, medication histories, and clinical variables into structured representations of patient health. Structured representations also arise in environmental monitoring datasets (e.g., NOAA, OpenAQ), where measurements are organized as tabular observations across space and time, often combined with statistical or machine learning models for large-scale exposure estimation [39].

Despite their heterogeneity, these datasets share several defining characteristics: moderate dimensionality relative to imaging or omics data, mixed variable types, and substantial missingness. These properties define a regime in which prediction is often performed directly over heterogeneous variables, typically without relying primarily on large-scale representation learning.

From a modeling perspective, structured biomedical prediction can be viewed as learning predictive relationships over heterogeneous variables grouped across biological, clinical, behavioral, or environmental subsystems. At a schematic level, the input space can be represented as

(1) $$x=\left[x^{\left(1\right)},x^{\left(2\right)},\ldots ,x^{\left(K\right)}\right],$$

where each $x^{\left(k\right)}$, for k=1,…,K, denotes a subset of variables associated with a particular modality, representation level, or subsystem. The corresponding predictive task can then be represented schematically as

(2) $$\hat{y}=f\left(x^{\left(1\right)},x^{\left(2\right)},\ldots ,x^{\left(K\right)}\right),$$

where *f* operates directly on structured or tabular inputs. In this regime, interactions across heterogeneous variable groups are typically captured implicitly through the learned predictive structure of *f* rather than through explicit mechanistic coupling or deeply hierarchical latent representations.

In real-world datasets, missingness is pervasive due to incomplete measurements, irregular acquisition, and heterogeneous data collection processes. Partial observability can be represented schematically through subsystem-specific availability masks:

(3) $$\hat{y}=f\left(x^{\left(1\right)}⊙m^{\left(1\right)},\ldots ,x^{\left(K\right)}⊙m^{\left(K\right)}\right),$$

where ⊙ denotes element-wise masking and $m^{\left(k\right)}$, for k=1,…,K, indicates modality or variable availability. The ability to operate robustly under incomplete information is therefore a central practical requirement for models in this regime.

Table 5 and Table 6 reveal a highly consistent modeling pattern across datasets. Linear and generalized linear models dominate as the primary modeling approach, accounting for more than half of all studies (53.6%) and appearing in the majority of papers (57.3%). This dominance is particularly pronounced in population-based cohort datasets (ELSA, HRS, MIDUS, NHANES), where GLM-based approaches exceed 70–85% of primary models. This empirical pattern is consistent with prior systematic analyses, which show that logistic regression remains widely used and often competitive across diverse clinical prediction settings [40]. Earlier reviews of structured risk modeling report similar trends [41], and broader assessments of prediction model development likewise highlight the persistent centrality of regression-based approaches in medical decision support [42].

Tree-based ensemble methods represent the second major class of models, particularly in clinical datasets such as MIMIC and eICU, where they account for a large fraction of primary models and are present in more than half of the studies. Their widespread adoption reflects their ability to capture non-linear interactions in heterogeneous tabular data while maintaining robustness to missingness and feature scaling. This pattern is consistent with reviews of machine learning in EHR analysis, where tree-based models remain prominent despite increasing use of deep architectures [34]. Related clinical evaluations similarly report sustained competitiveness of ensemble approaches in structured medical prediction [43].

Deep learning models are present across datasets but play a secondary role, accounting for a relatively small proportion of primary models (11.9%), despite appearing frequently in comparative analyses (44.7% prevalence). This gap between prevalence and primary usage suggests that deep learning is often evaluated alongside classical methods but less frequently selected as the final modeling approach. Similar patterns have been observed in large-scale evaluations of structured clinical data [40]. More broadly, recent analyses of tabular machine learning have argued that tree-based models often remain difficult for deep architectures to outperform in moderate-sized structured settings [44].

Within this framework, different model families correspond to different instantiations of the function *f*. Linear and GLM-based models provide interpretable and often well-calibrated predictions, which has contributed to their continued central role in epidemiological and clinical risk modeling [45]. Systematic reviews of prediction modeling have likewise highlighted the competitiveness and persistence of regression-based approaches in many structured settings [40], while similar conclusions emerge from broader evaluations of clinical risk models and prediction model methodology [41,42]. Tree-based ensemble methods capture higher-order interactions and are widely used in clinical prediction settings, including risk stratification and outcome prediction from EHR data [46]. In environmental applications, related ensemble-based approaches are used to model complex relationships between covariates and exposure variables at large spatial and temporal scales [39].

Overall, structured biomedical prediction provides a robust and interpretable foundation for biomedical modeling. However, its reliance on explicit variables and implicit interaction modeling can limit its ability to capture complex multimodal and heterogeneous relationships, motivating the transition toward higher-dimensional and multimodal learning regimes.

### 3.4. High-Dimensional Biomedical Signals

While structured data dominate population-level modeling, a distinct regime arises in the analysis of high-dimensional biomedical signals, including medical imaging, physiological waveforms, and omics data. Unlike tabular data, these modalities are not composed of predefined variables, but consist of raw measurements with complex spatial, temporal, or molecular structure. As a result, the central challenge shifts from modeling relationships between explicit features to extracting meaningful representations directly from high-dimensional inputs.

At a schematic level, high-dimensional biomedical prediction can be represented as a two-stage mapping involving representation learning followed by downstream prediction:

(4) $$z=\phi \left(x\right),\;\hat{y}=g\left(z\right),$$

where *x* denotes a raw high-dimensional input such as an image, physiological waveform, or omics profile, ϕ is a learned representation function that transforms the input into a latent embedding *z*, and *g* is a downstream predictive function operating on that representation. In contrast to structured tabular prediction, the primary modeling challenge in this regime typically lies in learning informative and transferable representations through ϕ rather than in specifying the form of the downstream predictor itself.

Despite the diversity of modalities, Table 7 and Table 8 reveal a substantial shift in modeling paradigms relative to structured data, but not a uniform transition to deep learning dominance. Instead, the high-dimensional regime appears bifurcated. In the primary-model analysis (Table 7), linear and generalized linear models constitute the largest model family overall (35.5%), followed by deep learning (29.9%). This indicates that, even in high-dimensional settings, representation-learning approaches have not displaced classical methods universally.

The distribution is strongly modality-dependent. In imaging datasets, particularly CAMELYON, deep learning clearly dominates as the primary modeling approach (84.7%), consistent with the central role of convolutional architectures in digital pathology and medical imaging [47]. Foundational work on deep convolutional networks has established their effectiveness in learning hierarchical representations from image data [36]. ADNI shows a more mixed pattern, with deep learning (28.9%), linear models (27.5%), and kernel methods (18.8%) all contributing substantially, suggesting methodological pluralism rather than a single dominant paradigm.

Medical imaging provides the canonical instantiation of this framework. Convolutional neural networks learn hierarchical spatial representations ϕ(x) directly from pixel data, enabling accurate classification and detection in radiology and pathology [35]. Similar approaches have achieved expert-level performance in ophthalmology screening tasks [48]. High-dimensional physiological signals such as electrocardiograms can likewise be modeled using deep architectures that learn representations directly from raw waveforms [49], although their temporal structure introduces additional challenges that are discussed in Section 3.6. These examples illustrate how learned representations ϕ can capture clinically meaningful structure that is difficult to encode through manually engineered features.

In contrast, omics-oriented datasets exhibit a markedly different profile. In GTEx, linear and generalized linear models account for 61.1% of primary models, while survival models represent a further 19.4%. TCGA shows a similar pattern, with linear/GLM (39.0%) and survival models (29.0%) together exceeding deep learning by a wide margin. UK Biobank imaging-derived analyses also show a substantial role for linear models (50.5%), exceeding deep learning (23.3%). In these settings, the representation function ϕ is often constrained by limited sample size and high measurement noise, and is frequently implemented through dimensionality reduction or regularization rather than deep architectures. Reviews of deep learning in genomics and omics analysis report similar limitations under such conditions [50].

The prevalence analysis in Table 8 conveys a complementary perspective. Deep learning appears in 67.0% of studies overall, substantially exceeding its primary-model share, indicating that it is widely used even when not selected as the principal model. This gap between prevalence and primary usage suggests that deep learning frequently appears in comparative benchmarking pipelines, feature extraction stages, or hybrid workflows rather than serving as the sole decision-driving model.

Taken together, these results suggest that the defining feature of this regime is not universal deep learning dominance, but coexistence between two modeling paradigms: representation-driven deep learning, which dominates image-centric problems, and statistically grounded models, which remain central in many omics and biomedical signal applications. This interpretation is consistent with broader reviews showing that deep learning has transformed imaging tasks [47], while more heterogeneous high-dimensional biomedical domains continue to rely heavily on classical statistical and survival modeling approaches [50].

A defining property of this regime is its dependence on data scale and quality. The estimation of ϕ requires sufficient coverage of the underlying data distribution, making deep learning models sensitive to dataset size, annotation quality, and distributional shifts. This contrasts with structured models, which can perform well in moderate-sample settings.

In addition to statistical constraints, practical considerations further limit deployment. Imaging and omics data require specialized acquisition equipment, standardized protocols, and substantial computational resources. Consequently, while high-dimensional models can provide detailed and often mechanistically informative representations, their applicability is typically restricted to controlled clinical or research environments.

Overall, high-dimensional modeling shifts the focus from feature engineering to representation learning, enabling powerful modeling of complex biological signals. However, this increased expressivity comes at the cost of higher data requirements and reduced deployability, reinforcing the complementary role of structured and high-dimensional approaches in biomedical machine learning.

### 3.5. Multimodal Integration

While the previous sections considered structured variables and high-dimensional signals largely in isolation, real-world biomedical prediction problems often require their integration into unified predictive systems. Multimodal machine learning addresses this problem by combining heterogeneous sources of information—such as clinical records, imaging, physiological signals, behavioral surveys, and environmental measurements—to improve predictive performance and capture interactions across levels of organization [8].

A central challenge in this setting is the heterogeneity of input spaces. Modalities differ not only in dimensionality and structure, but also in noise characteristics, sampling frequency, alignment, and semantic meaning. Imaging encodes spatial patterns, physiological signals capture temporal dynamics, while structured clinical or behavioral variables summarize aggregated or subjective measurements. Effective multimodal integration therefore requires models that reconcile these differences while preserving modality-specific information.

At a schematic level, multimodal learning can be viewed as integrating heterogeneous inputs originating from multiple measurement domains or sensing modalities. Let

(5) $$x=\left[x^{\left(1\right)},x^{\left(2\right)},\ldots ,x^{\left(M\right)}\right],$$

where each $x^{\left(m\right)}$ corresponds to a distinct modality such as imaging, omics, physiological signals, clinical records, or behavioral data. For high-dimensional modalities, inputs are typically transformed into learned latent representations

(6) $$z^{\left(m\right)}=\phi^{\left(m\right)}\left(x^{\left(m\right)}\right),$$

through modality-specific encoders, while structured modalities may be incorporated directly or through shallow transformations. Multimodal prediction can then be represented schematically as

(7) $$\hat{y}=f\left(z^{\left(1\right)},z^{\left(2\right)},\ldots ,z^{\left(M\right)}\right),$$

where *f* denotes a fusion mechanism integrating information across modalities. Depending on the architecture, this integration may occur through early fusion, intermediate latent-space interaction, attention-based alignment, or late-stage decision fusion. The central modeling challenge in this regime is therefore not only representation learning within individual modalities, but also preserving complementary information and cross-modal relationships during integration.

Table 9 and Table 10 reveal that, unlike the structured regime where one model family clearly dominates, multimodal modeling is characterized by substantial methodological fragmentation. In the primary-model analysis, linear and generalized linear models constitute the largest family overall (29.1%), followed by deep learning (22.5%) and tree-based ensembles (17.0%). This distribution suggests that multimodal integration is not governed by a single dominant modeling paradigm, but instead reflects multiple competing strategies for combining heterogeneous information sources.

This heterogeneity is strongly modality- and application-dependent. In omics-rich multimodal settings such as GTEx and TCGA, linear and survival models remain prominent. In contrast, digital phenotyping and behavioral sensing datasets such as StudentLife, mPower, and RADAR-CNS show larger roles for deep learning and tree-based ensembles. UK Biobank multimodal studies exhibit a mixed profile, with linear models, deep learning, and ensembles all contributing substantially. Collectively, these patterns suggest that multimodal learning often inherits modeling tendencies from constituent modalities rather than replacing them with a unified “multimodal-specific” model class.

The prevalence analysis conveys a complementary perspective. Deep learning appears in 74.2% of studies overall, far exceeding its 22.5% share as the primary model family. This gap indicates that deep architectures are pervasive components of multimodal pipelines—even when they are not the final decision-driving model. In many studies, deep learning appears as a modality-specific encoder, feature extractor, or component within hybrid systems. Reviews of multimodal learning report similar patterns, emphasizing that deep representation learning is often foundational to multimodal pipelines without necessarily defining the final predictive model [8]. Related work on multimodal sequence modeling further highlights the role of deep architectures as intermediate representation learners rather than standalone predictors [51].

Taken together, these results suggest that the defining characteristic of this regime is not deep learning dominance per se, but coexistence between representation-driven fusion models and hybrid predictive architectures that combine classical and modern machine learning components. This aligns with broader observations that multimodal systems are often organized around integration strategies rather than a single model class [8].

**Figure 3:**
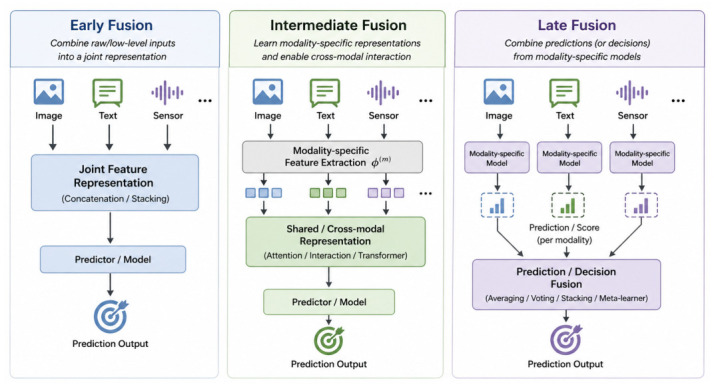
Early, intermediate, and late multimodal fusion strategies, showing integration at the input, representation, and decision levels.

Within this framework, different multimodal architectures correspond to different choices of the fusion function *f* and the stage at which representations are combined. Early fusion combines raw or low-level features into a joint representation prior to prediction [37], late fusion combines modality-specific predictions at the decision level [52], while intermediate fusion learns modality-specific embeddings that interact through shared latent spaces or attention mechanisms [53]. Transformer-based architectures extend this approach by enabling cross-modal interactions through learned attention across modalities [51]. Figure 3 summarizes these fusion strategies and highlights their structural differences, providing a visual reference for the trade-offs discussed below.

These strategies reflect different trade-offs between expressivity and robustness. Early fusion can capture rich cross-modal interactions but often requires aligned and fully observed inputs. Late fusion is typically more robust to missing modalities and facilitates modular development, but may underutilize deeper cross-modal dependencies. Intermediate fusion attempts to balance these properties and has become increasingly prominent in modern multimodal architectures.

A defining practical challenge of multimodal systems is incomplete modality availability during training or deployment. At a schematic level, modality availability can be represented through binary indicators $a^{\left(m\right)}\in 0,1$:

(8) $$\hat{y}=f\left(a^{\left(1\right)}z^{\left(1\right)},\ldots ,a^{\left(M\right)}z^{\left(M\right)}\right),$$

where $a^{\left(m\right)}=0$ indicates that modality *m* is unavailable. Unlike conventional missing values in structured tabular data, missing modalities remove entire representational channels, creating a qualitatively different robustness and integration problem.

Multimodal systems may also involve temporally asynchronous or irregularly sampled measurements across modalities. At a schematic level, such observations can be represented as

(9) $$x={\left\{\left(x_{t_{i}}^{\left(m\right)},t_{i}\right)\right\}}_{i=1}^{N_{m}},$$

where observation times $t_{i}$ for modality *m* are generally nonuniform and may differ across modalities. This creates additional challenges involving temporal alignment, synchronization, and cross-modal consistency.

This challenge has motivated approaches such as modality dropout, latent-space imputation, and adaptive fusion mechanisms that dynamically reweight available modalities [11]. The methodological diversity observed in Table 9 and Table 10 likely reflects these competing strategies, as no single fusion approach is universally robust.

Beyond statistical considerations, multimodal integration introduces substantial practical constraints. Modalities differ widely in acquisition cost, invasiveness, temporal availability, and scalability, making the simultaneous modality availability assumed in many multimodal architectures unrealistic in real-world deployment settings. This creates a recurring tension between predictive expressivity and deployability, particularly in clinical or large-scale screening environments where only subsets of modalities may be available at inference time.

At a schematic level, this setting can be represented as prediction over dynamically varying modality subsets:

(10) $$\hat{y}=f_{S}\left(z^{\left(m\right)}:m\in S\right),\;S\subseteq 1,\ldots ,M,$$

where *S* denotes the subset of modalities available for a given sample or deployment context. In practice, the predictive function may therefore operate under multiple modality configurations rather than under a single fixed multimodal input space. Designing systems that remain robust across heterogeneous and partially observed modality subsets remains a central challenge in deployable biomedical machine learning.

Overall, multimodal integration represents one of the most expressive regimes in biomedical machine learning, not because it is dominated by a particular model family, but because it explicitly treats integration itself as the modeling problem. At the same time, this expressivity introduces additional challenges related to modality heterogeneity, incomplete observation, and system complexity, positioning multimodal learning at the center of the trade-offs that define deployable biomedical systems.

### 3.6. Temporal and Longitudinal Modeling

Many phenomena in the biological and behavioral sciences evolve over time, making temporal and longitudinal modeling a fundamental component of biomedical machine learning. Unlike static prediction settings, where inputs represent a single snapshot, temporal modeling requires capturing trajectories, temporal dependencies, and the evolution of risk across multiple time scales.

At a schematic level, temporal and longitudinal modeling can be viewed as learning predictive relationships over sequences of observations evolving across time. Let

(11) $$x={x_{t}}_{t=1}^{T},\;x_{t}\in X$$

denote a temporally ordered sequence of observations, where each $x_{t}$ corresponds to the system state or measurement set available at time *t*. The associated predictive task can then be represented schematically as

(12) $$\hat{y}=f\left(x_{1},x_{2},\ldots ,x_{T}\right),$$

where *f* models temporal dependencies, trajectories, or state transitions across the sequence. Depending on the application, *f* may represent recurrent architectures, temporal convolutional models, transformers, continuous-time systems, or probabilistic temporal processes.

In heterogeneous biomedical settings, each temporal observation $x_{t}$ may itself contain heterogeneous variables, subsystem-level measurements, or modality-specific latent representations, linking temporal modeling naturally to both structured and multimodal formulations. As a result, temporal models often operate simultaneously across heterogeneous biological, clinical, behavioral, and environmental representations while also accounting for temporal evolution and irregular observation dynamics.

Table 11 reveals that temporal modeling does not correspond to a single dominant “sequence modeling” paradigm, but instead spans two distinct methodological traditions. In the primary-model analysis, linear and generalized linear models remain the largest family overall (42.3%), substantially exceeding deep learning (12.7%) and tree-based ensembles (16.2%). This indicates that temporal modeling in biomedical and behavioral science remains largely rooted in statistical longitudinal modeling, despite growing interest in recurrent and transformer-based architectures.

This pattern is strongly dataset-dependent. Longitudinal cohort studies such as ELSA, HRS, MIDUS, and NHANES are dominated by linear and GLM-based approaches, which account for a large majority of primary models. Survival models also play a substantial role in these settings, reflecting a focus on time-to-event outcomes and repeated-measures inference. This is consistent with the long-standing centrality of survival analysis and related statistical frameworks in biomedical research [54].

In contrast, dense clinical and digital sensing datasets exhibit a different profile. In MIMIC, tree-based ensembles and deep learning models play larger roles, while mPower and StudentLife show increased prominence of deep learning and ensemble approaches. Environmental datasets such as OpenAQ also show substantial contributions from both deep learning and tree-based methods, while NOAA exhibits strong use of ensemble approaches. These settings support higher-frequency observations and often involve forecasting or continuous monitoring tasks, favoring more flexible non-linear models, as also observed in large-scale clinical time-series benchmarks [55].

The prevalence analysis provides a complementary perspective. Linear and GLM-based models appear in 52.0% of studies overall, while deep learning appears in 47.1%, substantially exceeding its primary-model share. This gap suggests that deep temporal models are frequently explored within comparative or hybrid pipelines, even when not selected as the final model. Reviews of machine learning for longitudinal and time-series healthcare data report similar patterns, where deep architectures expand modeling flexibility but often complement rather than replace classical statistical approaches [34]. Deep sequence models such as LSTMs have been successfully applied to clinical time-series prediction tasks [56], alongside alternative architectures such as temporal convolutional networks that capture long-range dependencies with improved stability [57].

Taken together, the temporal regime appears bifurcated. One subregime is dominated by statistical longitudinal modeling, particularly in sparse observational cohorts. The other centers on data-driven sequence modeling in dense clinical and sensing data. This dual structure distinguishes temporal modeling from both structured and high-dimensional regimes and reflects fundamental differences in data resolution and sampling.

Within this framework, different modeling approaches correspond to different instantiations of the temporal function *f*. Classical statistical models specify temporal structure explicitly through parametric assumptions, while sequence models attempt to learn these dependencies directly from data. Recurrent neural networks, LSTMs, temporal convolutional networks, and transformer-based models provide increasingly flexible implementations of *f* for dense temporal signals, including attention-based architectures such as the Temporal Fusion Transformer [58]. At the same time, the empirical distributions in Table 11 and Table 12 suggest that such architectures complement rather than replace classical models in many domains.

A central practical constraint in longitudinal biomedical modeling is irregular sampling. In many real-world datasets, observations occur at uneven and nonuniform intervals rather than at fixed periodic time points. At a schematic level, irregularly sampled observations can be represented as

(13) $$x={\left\{\left(x_{t_{i}},\;t_{i}\right)\right\}}_{i=1}^{N},$$

where measurements $x_{t_{i}}$ are observed at generally irregular time points $t_{i}$. This setting violates the assumptions of many standard sequence models that implicitly rely on regularly spaced temporal observations. Missingness may additionally occur both within and across time points, further complicating estimation of temporal dependencies and latent dynamics.

Recent approaches such as continuous-time latent variable models and neural differential equations explicitly attempt to address this issue by modeling temporal evolution in continuous time rather than through fixed discrete sampling assumptions [59]. These constraints may help explain why simpler longitudinal models remain prominent across many biomedical datasets despite advances in deep temporal modeling.

A further challenge arises when multiple modalities evolve at different temporal resolutions. Behavioral variables may be measured annually, clinical variables episodically, and physiological signals continuously. Temporal alignment across modalities is therefore often imperfect, requiring aggregation, interpolation, or asynchronous modeling strategies. Such transformations can simplify the problem but may obscure important dynamic relationships.

These constraints highlight a key distinction between dense clinical time series and sparse longitudinal cohorts. The former support rich sequence modeling, whereas the latter often favor hybrid approaches that embed temporal summaries within otherwise structured models. The persistence of GLM and survival dominance suggests that much temporal modeling in practice remains closer to structured prediction augmented with longitudinal information than to end-to-end sequence modeling.

Consequently, the choice of temporal modeling strategy is governed not simply by whether data are longitudinal, but by temporal resolution, regularity, and alignment across modalities. In many real-world settings these constraints limit the practical deployment of highly expressive sequence models, favoring simpler temporal representations that balance predictive performance, robustness, and data availability.

Overall, temporal modeling extends biomedical machine learning from static prediction to dynamic processes, but does so through a diverse methodological landscape rather than a single dominant model family. This diversity reflects the fundamentally heterogeneous nature of temporal data.

### 3.7. Cross-Regime Empirical Synthesis

**Figure 4:**
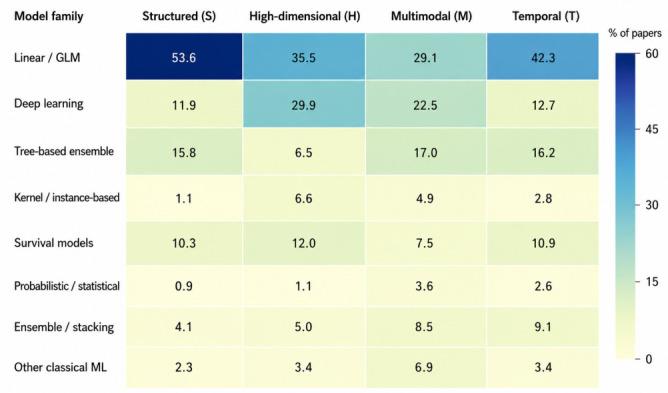
Dominant model-family distributions across prediction regimes. Values are within-regime percentages.

Although individual datasets and prediction settings exhibit substantial methodological heterogeneity, several broad cross-regime patterns emerge from the dominant-model analyses summarized throughout Table 5, Table 6, Table 7, Table 8, Table 9, Table 10 and Table 11. Figure 4 provides a compact synthesis of these trends by comparing the relative prevalence of dominant model families across the structured, high-dimensional, multimodal, and temporal prediction regimes.

The strongest overall pattern is the continued prominence of linear and generalized linear models across all four prediction regimes. Even in settings involving high-dimensional or multimodal biomedical data, classical statistical model families remain highly represented within the retrieved literature. This likely reflects a combination of factors, including interpretability, compatibility with moderate cohort sizes, established biomedical workflows, and the continued importance of tabular and structured variables across many health datasets.

Deep learning exhibits a different empirical pattern, appearing more prominently in the high-dimensional and multimodal regimes than in the structured or temporal settings. This is consistent with the broader role of representation learning in imaging, signal-processing, and heterogeneous modality-integration tasks. However, the retrieved literature does not suggest a uniform replacement of classical approaches by deep learning. Instead, deep architectures appear alongside statistical, ensemble, and hybrid modeling strategies across many datasets and prediction settings.

Tree-based ensemble methods remain strongly represented in the structured, multimodal, and temporal regimes, while appearing less frequently as dominant models in the high-dimensional regime. This may reflect the continued effectiveness of ensemble approaches for heterogeneous tabular and mixed-feature biomedical datasets. Survival-oriented methods also remain visible across multiple regimes rather than being confined exclusively to longitudinal studies, suggesting that time-to-event and progression-oriented prediction problems continue to influence a broad range of biomedical machine-learning applications.

Taken together, the heatmap reinforces the broader empirical interpretation developed throughout the regime-specific analyses: machine-learning usage patterns vary substantially across prediction settings, but the retrieved literature does not support a simple transition from classical statistical approaches toward universally dominant deep-learning paradigms. Instead, the observed ecosystem remains methodologically mixed, with different regimes emphasizing different combinations of model families depending on data structure, modality composition, and prediction objectives.

### 3.8. Learning Under Missing and Partial Modalities

**Figure 5:**
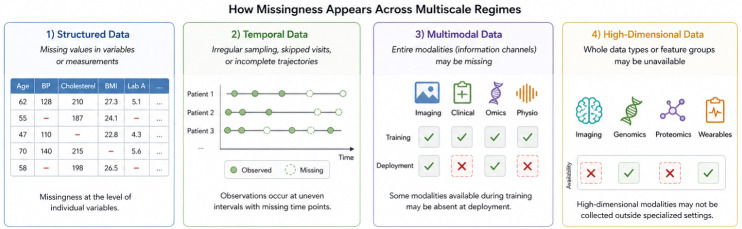
Missingness across heterogeneous biomedical prediction regimes, including missing values in structured data, irregular temporal sampling, missing modalities, and unavailable high-dimensional data types.

A recurring theme across the prediction regimes reviewed above is that heterogeneous biomedical data are rarely complete. In structured datasets, missingness appears at the level of individual variables or measurements. In high-dimensional settings, entire data types such as imaging or omics may be unavailable. In multimodal systems, missingness affects whole information channels, while in temporal settings it appears as irregular sampling or incomplete trajectories. Missingness is therefore not a secondary preprocessing issue, but a defining property of real-world biomedical prediction settings [60]. Figure 5 summarizes how different forms of missingness arise across heterogeneous biomedical prediction regimes, including variable-level, temporal, modality-level, and high-dimensional data gaps.

This challenge can be represented schematically by extending the multimodal formulation to account for partial modality availability. Let $z^{\left(m\right)}$ denote the representation associated with modality *m*, and let $a^{\left(m\right)}\in \left\{0,1\right\}$ indicate whether that modality is available for a given sample or deployment setting. Prediction under partial observability can then be represented as

(14) $$\hat{y}=f\left(a^{\left(1\right)}z^{\left(1\right)},\ldots ,a^{\left(M\right)}z^{\left(M\right)}\right).$$

At a practical level, this formulation highlights that missingness may occur not only within individual feature vectors, but also at the level of entire modalities, thereby removing complete representational channels rather than isolated measurements. As a result, multimodal systems must often operate under heterogeneous and dynamically varying information availability conditions.

The manifestation of this problem differs across regimes. In structured prediction, missing values are typically handled through imputation, missingness indicators, or models that tolerate incomplete covariates. Classical approaches to missing data, including multiple imputation and likelihood-based methods, remain widely used in these settings [61]. In high-dimensional modeling, the issue is often more fundamental: modalities such as imaging or omics may not be available outside specialized settings, limiting their applicability at deployment. In temporal modeling, missingness is coupled to time, producing irregular sequences and variable follow-up intervals that violate assumptions of standard sequence models [62]. In multimodal systems, training data may include multiple modalities, while deployment may involve only a subset.

Traditional imputation methods are effective when missingness occurs within a modality, but are insufficient when entire modalities are absent. Models trained on rich multimodal inputs may implicitly depend on modalities that are expensive, invasive, or impractical to collect, creating a deployment mismatch between training-time and inference-time information availability. This problem is more accurately understood as a partial observability or modality-coverage problem rather than classical distribution shift in the statistical sense. By contrast, distribution shift refers to changes in the underlying data distribution across populations, environments, acquisition protocols, or deployment settings. In practice, biomedical systems may encounter both incomplete modality availability and distribution shift simultaneously, and both can substantially degrade predictive performance under real-world deployment conditions [63].

Recent approaches attempt to address this challenge by explicitly modeling partial modality availability. Modality dropout introduces stochastic removal of inputs during training to improve robustness [64]. Multimodal learning frameworks incorporate modality-specific encoders and flexible fusion mechanisms that operate on subsets of available inputs [8]. More recent methods explore adaptive multimodal fusion and missing-modality learning strategies intended to improve robustness under incomplete data availability [11]. Related work has also investigated staged teacher–student and distillation-based approaches that transfer predictive structure learned from richer longitudinal training settings into deployment-constrained biomedical screening models operating under reduced feature availability and practical deployment constraints [65].

However, missing modalities cannot always be treated as noise. Different modalities often carry complementary and non-redundant information. Removing a modality may therefore eliminate an entire class of biological, behavioral, or observational information from the model. For example, a system trained with both clinical and imaging data may not retain the same predictive meaning when imaging is absent; similarly, a longitudinal model trained on repeated measurements may behave differently when only baseline observations are available.

For this reason, modality availability should be treated as a first-class design constraint. Models should be evaluated not only under full-data conditions, but also under realistic deployment scenarios in which costly or difficult-to-collect modalities are absent. This perspective reframes missingness from a nuisance to be corrected into a central property of heterogeneous biomedical systems.

Overall, learning under missing and partial modalities connects the methodological regimes discussed above to deployment. Structured, high-dimensional, multimodal, and temporal models differ in how they represent data, but all face the same practical question: which parts of the heterogeneous biomedical input space will actually be available at inference time? Addressing this question is essential for moving from high-performing research models to deployable biomedical and behavioral systems.

### 3.9. From Models to Deployable Systems

The prediction regimes discussed above reveal a recurring tension in biomedical machine learning: the modalities and model architectures that maximize predictive performance are often the least compatible with real-world deployment. Structured models are generally accessible and interpretable but may underutilize complex multimodal relationships. High-dimensional and multimodal systems can capture richer biological and clinical representations, yet often depend on costly, invasive, or operationally difficult data sources. Temporal models may improve prediction through longitudinal context, but frequently assume data density unavailable in practical settings. Moving from models to deployable systems therefore requires treating deployment constraints, modality availability, and partial observability as integral components of the modeling problem itself [66].

At a schematic level, this tension can be represented as a mismatch between the information space available during model development and the information space available during real-world deployment. Let

(15) $${\hat{y}}_{\mathrm{train}}=f\left(z^{\left(1\right)},z^{\left(2\right)},\ldots ,z^{\left(M\right)}\right)$$

denote a predictive model trained using the full set of available modalities or representations. In deployment settings, however, only a subset of modalities may be accessible, leading instead to predictions of the form

(16) $${\hat{y}}_{\mathrm{deploy}}=f_{S}\left(\{z^{\left(m\right)}:m\in S\}\right),\;S\subseteq \left\{1,\ldots ,M\right\}.$$

Here, *S* denotes the subset of modalities available under a particular deployment condition. This formulation highlights that the assumptions under which a model is trained may differ substantially from those under which it ultimately operates, creating a deployment gap between development-time and inference-time information availability [10]. Similar concerns have been emphasized in healthcare applications, where differences between curated training data and real-world operational conditions can substantially affect reliability, robustness, and generalization [63]. Related work on dataset and distribution shift further illustrates how such mismatches may degrade predictive performance outside development environments [67].

Under this formulation, robustness to deployment constraints can be interpreted as approximate stability of predictive performance across varying modality subsets *S*. In practice, this motivates approaches such as modality dropout, adaptive fusion, distillation, and deployability-aware feature selection, all of which attempt to reduce dependence on any single modality while preserving sufficient predictive information under partial observability. The objective is therefore not merely maximizing performance under complete-information conditions, but maintaining reliable inference as the available information space changes between training and deployment.

This gap manifests across all prediction regimes. In structured settings, clinically derived or laboratory variables may improve performance but limit scalability. In high-dimensional modeling, imaging and omics provide rich representations while remaining impractical for large-scale screening. In multimodal systems, models may implicitly depend on combinations of modalities that are rarely co-observed in practice. In temporal settings, sequence models often assume longitudinal density unavailable in real-world monitoring. Across regimes, predictive performance is therefore constrained not only by modeling capacity, but by what can realistically be measured.

One approach to addressing this mismatch is to separate predictive richness during training from simplicity at inference. Knowledge distillation provides a formal mechanism for this separation, using a high-capacity teacher trained on rich multimodal biomedical inputs to supervise a student model restricted to deployable modalities [13]. In this framework, the goal is not merely to compress a model, but to transfer information from inaccessible modalities into a predictor that can operate under realistic constraints. This approach is particularly relevant in biomedical settings, where the most informative modalities are often the least scalable. More broadly, model compression and efficiency-aware learning have been studied as key enablers of practical deployment [68].

A complementary strategy is deployability-aware feature selection. Rather than beginning with a maximal multimodal system and compressing it afterward, one can explicitly optimize for predictive sufficiency under accessibility constraints, identifying subsets of behavioral, environmental, or routinely collected variables that preserve much of the predictive signal while remaining practical to obtain. This shifts the objective from maximizing accuracy alone to identifying representations of risk that are compatible with intended use.

These strategies highlight a broader view of machine learning systems in which models are only one component of deployment. Data acquisition burden, computational requirements, robustness to partial inputs, interpretability, and operational integration all influence whether a model can function outside controlled research settings. This systems perspective has been emphasized in the study of production machine learning, where non-algorithmic factors often dominate real-world performance [10]. Similar conclusions have been drawn in applied ML systems research, which emphasizes lifecycle considerations such as monitoring, feedback, and human–AI interaction [66].

Viewed through this lens, the central trade-off in biomedical machine learning is not classical versus deep learning, or unimodal versus multimodal modeling, but expressivity versus deployability. More expressive models leverage richer multimodal and cross-domain information but increase dependence on assumptions about data availability. Simpler models may sacrifice some predictive capacity yet provide robustness, accessibility, and scalability. The most effective systems often lie between these extremes, combining expressive training-time models with constrained inference-time models through distillation, modality reduction, or hybrid system design.

This perspective suggests that deployment should not be treated as a downstream implementation concern, but as an organizing principle for model development itself. Rather than asking how to deploy a model after it is built, a more consequential question is how to design models whose assumptions already reflect deployment realities. In this sense, the progression from structured prediction to multimodal and temporal learning is not only a progression in modeling sophistication, but also a progression toward jointly optimizing predictive performance and deployability.

## 4. Bridging Heterogeneous Biomedical Data and Machine-Learning Systems

The preceding analysis focused on how different data regimes give rise to distinct modeling approaches. A complementary question is how these components are assembled into coherent predictive systems. In practice, biomedical machine-learning systems are rarely defined by a single model. They are more often organized as structured pipelines that transform heterogeneous inputs into representations, predictions, and ultimately deployable decision processes.

This systems perspective has become increasingly prominent in biomedical AI, where performance depends not only on model choice, but on how information is organized, transferred, and constrained across stages of the pipeline [34]. Similar observations have been made in large-scale clinical AI systems, where architectural design and data flow can be as important as the predictive model itself [43]. More broadly, translational perspectives on machine learning emphasize that successful deployment requires integrating modeling with data acquisition, workflow, and operational constraints [1].

### 4.1. From Representations to System Architectures

Across heterogeneous biomedical prediction settings, predictive modeling can be viewed as a composition of representation learning and integration. In practice, however, these components are rarely implemented as a single monolithic function. Instead, they are distributed across pipelines that combine preprocessing, representation learning, integration, and prediction in structured and often modular ways.

A central challenge is that modalities remain fundamentally heterogeneous even after representation learning. Imaging, omics, clinical variables, physiological signals, and behavioral data encode different aspects of health using incompatible abstractions. As a result, integration is not merely a technical step, but a design decision that determines how cross-domain and cross-modality relationships are exposed, approximated, or suppressed [8].

Different integration strategies reflect different system-level assumptions. Early fusion emphasizes joint representation learning but requires aligned inputs, while late fusion promotes modularity and robustness by separating modality-specific predictors [52]. Intermediate and attention-based approaches attempt to balance these properties, enabling interaction across modalities without requiring strict alignment [51,53]. Temporal extensions introduce additional constraints, as representations must accommodate irregular sampling and evolving system states [56].

Taken together, these observations suggest that biomedical machine learning is best understood not as a collection of isolated models, but as a process of organizing transformations across heterogeneous data sources. The emphasis is therefore on representation, integration, information flow, and deployment constraints rather than on explicit mechanistic scale-coupling models.

### 4.2. Recurrent Pipeline Motifs Across Prediction Regimes

While individual models vary widely, many biomedical machine-learning systems exhibit recurring architectural organization patterns that extend beyond individual model families. To characterize these patterns, an auxiliary descriptive audit was performed over the OpenAlex corpus constructed for this review, focusing on pipeline-level structure rather than isolated predictive algorithms. The audit is intended as an exploratory organizational synthesis rather than as a formal systematic review or statistically validated meta-analysis.

For each paper, titles, abstracts, and when available extended text were parsed using rule-based patterns designed to detect architectural elements such as explicit preprocessing, learned embeddings, fusion mechanisms, staged prediction, and robustness strategies. Papers were then annotated with one or more motifs, allowing aggregation at the regime level. Because many systems contain overlapping architectural characteristics, the resulting categories should be interpreted as approximate organizational descriptors rather than mutually exclusive formal classes.

Five recurring motifs were identified, corresponding to common ways of organizing representation and prediction: handcrafted feature pipelines (explicit preprocessing and engineered variables), learned representation pipelines (latent embeddings learned from raw inputs), fusion-centric architectures (explicit cross-modal interaction mechanisms), sequential predictive pipelines (staged models where intermediate outputs feed downstream predictors), and robust or transfer-aware pipelines (systems explicitly incorporating mechanisms such as missing-modality handling, transfer learning, uncertainty-aware fusion, modality dropout, or deployment-aware adaptation). These motifs are not mutually exclusive, and many systems combine multiple design patterns.

**Figure 6:**
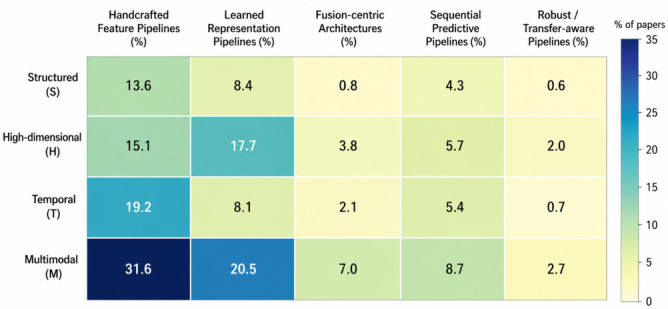
Prevalence of common pipeline architecture motifs across prediction regimes. Values indicate the percentage of papers in which each motif appears; motifs are not mutually exclusive.

Figure 6 summarizes the distribution of recurring pipeline motifs across prediction regimes and highlights several broad trends.

First, handcrafted pipelines remain common across all regimes, particularly in multimodal and temporal settings, reflecting the continued importance of preprocessing and domain-informed representations in applied biomedical modeling [34]. Second, learned representation pipelines become more prominent as data dimensionality and heterogeneity increase, consistent with the central role of representation learning in high-dimensional domains [36,47].

Fusion-centric architectures appear less frequently than might be expected from the multimodal literature, suggesting that many practical systems continue to rely on relatively shallow, modular, or implicit integration strategies rather than on highly specialized fusion mechanisms [8]. In contrast, sequential predictive pipelines are widely observed, particularly in temporal and multimodal contexts; these staged systems, in which intermediate outputs inform downstream models, align with broader trends toward modular and distillation-based architectures in machine learning systems [13].

Finally, robust or transfer-aware pipelines remain comparatively uncommon. This comparatively limited prevalence may reflect several characteristics of current biomedical machine-learning research, including the dominance of performance-oriented benchmark objectives, limited modality diversity in many datasets, deployment complexity, and the relatively recent emergence of techniques such as knowledge distillation, modality-invariant learning, and graceful-degradation architectures. In addition, robustness-oriented design choices are not always explicitly described in abstracts or metadata-accessible text. Despite increasing recognition of missing modalities and deployment constraints, such mechanisms still appear relatively infrequently in the surveyed corpora, consistent with prior discussions suggesting that deployment robustness and real-world reliability remain challenging considerations in biomedical AI development [63].

Overall, these findings suggest that modular pipeline organization remains common across the surveyed literature, particularly in multimodal and temporal settings, although the present audit should be interpreted as descriptive rather than exhaustive. This observation is consistent with prior analyses of clinical and multimodal systems, which emphasize the importance of architectural organization and information flow alongside model choice [1,43].

### 4.3. Design Trade-Offs in Biomedical Machine-Learning Systems

The architectural motifs above give rise to a set of recurring trade-offs that govern biomedical machine-learning system design.

A first trade-off concerns *modality richness versus accessibility*. Systems that incorporate richer modality sets can achieve higher predictive performance, but may be difficult to deploy when those modalities are unavailable in practice.

A second trade-off concerns *expressivity versus robustness*. Highly expressive models can capture complex multimodal and heterogeneous relationships, but are more sensitive to missing inputs and distribution shift, while simpler systems often provide greater stability under real-world variability [63].

A third trade-off concerns *integration depth versus flexibility*. Deep integration can exploit rich dependencies but typically assumes complete and aligned inputs, whereas modular integration provides greater resilience under partial observability [11].

Finally, there is a trade-off between *information preservation and tractability*. Rich representations improve fidelity but increase computational and statistical burden, while compressed representations improve efficiency at the cost of potential information loss.

These trade-offs are not independent, and system design requires balancing them jointly rather than optimizing any single dimension.

### 4.4. Toward Deployable Biomedical Systems

Taken together, these observations suggest that biomedical machine learning is fundamentally a systems-design problem. The challenge is not only to construct accurate predictive models, but to organize representations, integration mechanisms, and decision processes in a way that remains compatible with real-world constraints [69].

In this view, the deployment gap is not merely a downstream issue, but an architectural consequence of how systems are constructed. Dependence on specific modalities, assumptions about data completeness, and tightly coupled integration strategies can all limit the applicability of otherwise high-performing models.

Addressing this challenge requires design strategies that explicitly incorporate deployment constraints. These include modular pipelines that degrade gracefully under missing inputs, staged prediction systems that separate representation learning from decision-making, and hybrid architectures that combine expressive training-time models with constrained inference-time components [10].

More broadly, bridging heterogeneous biomedical data and machine-learning systems is not simply a matter of combining modalities, but of explicitly structuring transformations across heterogeneous biomedical representations so that relevant information is preserved under the constraints of real-world use. In this view, robustness, interpretability, and deployability are not downstream considerations, but properties that must be encoded directly into system design, a perspective that aligns with recent efforts to translate machine learning into real-world clinical and operational settings [70].

These deployment challenges increasingly emerge across real-world biomedical AI systems involving heterogeneous, incomplete, and dynamically evolving data streams. More concrete representative application scenarios are discussed in the following subsection.

### 4.5. Representative Application Scenarios

The methodological regimes discussed throughout this review become particularly relevant when viewed through concrete biomedical deployment settings. Although many systems combine multiple regimes simultaneously, several representative scenarios illustrate how different modeling constraints emerge in practice.

In intensive care prediction systems, such as those developed using MIMIC or eICU cohorts, models often integrate structured clinical variables, laboratory trajectories, physiological time series, and occasionally imaging data under conditions of incomplete and asynchronous observation. These settings emphasize robustness to missingness, temporal irregularity, and deployment constraints in continuously evolving clinical environments [43].

In multimodal oncology and precision medicine, machine learning systems increasingly combine histopathology, radiology, genomic profiling, and clinical variables to support diagnosis, prognosis, and treatment stratification. Such systems highlight the trade-off between predictive expressivity and real-world deployability, as many modalities remain expensive, invasive, or inconsistently available across institutions and patient populations [1].

Wearable and mobile-health systems provide another important multimodal biomedical setting in which behavioral, physiological, and environmental information may be integrated through smartphones, wearable sensors, and longitudinal digital phenotyping platforms. These applications introduce additional challenges involving noisy sensing, heterogeneous sampling frequencies, limited computational resources, and partial modality availability during real-world operation [27].

Taken together, these scenarios reinforce a central theme of this review: biomedical machine learning is shaped not only by predictive objectives, but by constraints related to modality availability, temporal structure, robustness, scalability, and deployment feasibility.

## 5. Open Challenges and Future Directions

The preceding sections suggest that progress in biomedical machine learning across heterogeneous data representations will depend less on isolated new algorithms than on systems that jointly address representation, integration, robustness, interpretability, and deployment. Current practice remains shaped by modular pipelines, partial observability, and constraints on data availability. These observations point to several open challenges that cut across prediction regimes.

### 5.1. Representing Heterogeneous Biomedical Structure

A central open question is how biological, clinical, behavioral, and environmental processes should be represented in a common computational framework. Current approaches often rely either on engineered variables that preserve interpretability or on learned embeddings that improve flexibility but may obscure scientific meaning. Future work will need representations that are not only predictive, but also capable of preserving relationships across levels of organization.

Foundation models and self-supervised learning offer promising directions for reusable biomedical representations [71,72]. Graph-based and causal representation learning may also help encode biological hierarchy, interaction structure, and mechanisms rather than only statistical association [73,74]. Related directions involving multitask learning, transfer learning, and foundation-model adaptation may further support representation sharing across heterogeneous biomedical tasks, modalities, and populations [75,76,77]. Data-centric AI perspectives additionally emphasize that progress may depend not only on larger architectures, but also on improving data quality, annotation consistency, dataset curation, and representation coverage across heterogeneous biomedical populations and sensing environments [78]. A key future direction is therefore the development of representations that are simultaneously expressive, interpretable, transferable across tasks and populations, and robust to variability in real-world biomedical data acquisition.

### 5.2. Learning Under Partial Observability

Incomplete data remain one of the defining barriers to deployable biomedical machine-learning systems. Missing values, missing modalities, irregular temporal sampling, and deployment-time modality gaps all violate the assumptions of models trained under idealized data availability. Although imputation, modality dropout, adaptive fusion, and distillation provide useful strategies, robust or transfer-aware architectures remain relatively uncommon in the audited literature.

Future work should treat partial observability as a primary design condition rather than as a preprocessing problem. This includes models that adapt to varying modality subsets, quantify uncertainty when information is absent, and degrade gracefully when data sources are missing. Recent work on generative imputation and probabilistic time-series imputation illustrates how missingness can be modeled directly rather than merely repaired after preprocessing [79,80]. The broader challenge is to move from models trained on complete data with robustness added afterward toward models designed from the outset for incomplete-information settings.

Federated and privacy-preserving learning represent an additional important direction in this context, particularly for multi-institutional biomedical systems in which data-sharing constraints prevent centralized aggregation of sensitive patient information [81,82]. Such approaches may become increasingly important for biomedical machine learning across distributed clinical, behavioral, and sensing infrastructures while preserving privacy, governance, and regulatory constraints.

### 5.3. Alignment, Generalization, and Distribution Shift

Heterogeneous biomedical data are difficult to align because different processes unfold at different temporal, spatial, and biological scales. Molecular measurements may be sparse, clinical records episodic, behavioral data irregular, and sensing streams continuous. Current solutions often rely on aggregation, interpolation, or synchronization heuristics, but these can obscure important dynamic relationships.

Generalization adds a second layer of difficulty. Biomedical data vary across populations, institutions, devices, protocols, and environments, and multimodal biomedical models may amplify such differences when they rely on fragile cross-modal correlations. Benchmarks of real-world distribution shift show that performance can degrade substantially outside development settings [83]. Future research should therefore focus on alignment-aware and shift-aware modeling, including continuous-time models, hierarchical temporal representations, domain adaptation, and external validation under realistic deployment conditions. Uncertainty estimation is also central, since calibrated uncertainty can help identify when model predictions should not be trusted under shift [84].

Related concerns involving fairness, demographic bias, and clinical equity are also becoming increasingly important as biomedical machine-learning systems are deployed across heterogeneous populations and healthcare environments [43,85]. Distribution shift, incomplete representation of minority populations, and unequal data quality may propagate or amplify disparities across predictive pipelines. Future work should therefore integrate fairness-aware evaluation, bias mitigation, and uncertainty-aware deployment into the design and validation of biomedical machine-learning systems. Federated and privacy-preserving learning may additionally help support large-scale heterogeneous biomedical machine-learning systems while reducing barriers associated with centralized clinical data sharing [81,82].

### 5.4. Deployment-Aware System Design

A recurring conclusion of this review is that predictive performance and deployability are often in tension. Rich multimodal systems may perform well in research settings but depend on modalities that are expensive, invasive, or unavailable in scalable applications. Conversely, deployable systems often require constrained input sets, modular pipelines, and robust inference under partial information.

Future work should make deployment constraints part of model design rather than downstream implementation. Promising directions include teacher–student learning, modality reduction, deployability-aware feature selection, and hybrid systems that use rich training-time information while requiring only accessible inference-time inputs. This also requires better reporting and early-stage evaluation of AI systems in clinical settings, as emphasized by recent AI-specific reporting guidelines [86,87]. The central question is not simply how to deploy existing models, but how to build models whose assumptions already match the conditions under which they will be used.

More broadly, causal and counterfactual modeling may become increasingly important for deployment-aware biomedical AI systems that aim not only to predict outcomes, but also to support intervention-oriented reasoning and decision-making under uncertainty [88,89]. Related directions involving neural surrogate models, physics-informed learning, and hybrid mechanistic–machine-learning systems may further enable hybrid approaches combining biomedical machine learning with physiological modeling while preserving stronger links to biological or physical structure [90,91,92].

Although explicit mechanistic multiscale frameworks from systems biology and computational physiology remain outside the primary scope of the present review, future interaction between mechanistic modeling and biomedical machine-learning systems may become increasingly important for physiological simulation, personalized medicine, and intervention-aware decision support.

Foundation biomedical models may further reshape deployment-oriented system design by enabling transferable pretrained representations spanning imaging, clinical records, molecular data, physiological signals, and multimodal biomedical corpora [71,72]. However, these systems also introduce additional challenges involving computational scale, interpretability, fairness, robustness, and adaptation under deployment constraints.

### 5.5. Evaluation, Interpretability, and Scientific Use

Evaluation standards must also evolve. Many benchmarks emphasize discrimination under curated conditions, but deployable biomedical machine-learning systems should be tested under missing modalities, noisy measurements, temporal drift, population shift, and constrained operating conditions. Reporting only ideal-data performance risks overstating real-world utility. Calibration should therefore be treated as a core evaluation criterion, particularly for models intended to support decisions under uncertainty [93].

Interpretability remains equally important. Within heterogeneous and multimodal biomedical machine-learning systems, explanations may need to connect molecular measurements, physiological variables, clinical history, behavior, and environmental context across partially observed and temporally evolving representations. This requires more than local feature attribution; it requires representations and architectures whose structure supports meaningful integration, uncertainty awareness, and clinically interpretable information flow across heterogeneous data sources. In high-stakes settings, interpretable model design may be preferable to post hoc explanations of opaque models [9]. Future systems should therefore be evaluated not only by predictive accuracy, but also by robustness, calibration, interpretability, uncertainty awareness, practical usability, fairness, deployment reliability, and resilience under incomplete or shifting data conditions.

Taken together, these challenges suggest that biomedical machine learning is increasingly becoming a systems science of representation, integration, and deployment under constraints. The central goal is not merely to build more expressive models, but to design deployable biomedical systems that remain reliable, interpretable, and useful in the settings for which they are intended.

### 5.6. Limitations of the Present Review

Several limitations should be considered when interpreting the analyses presented in this review. First, the computational literature-mining framework relied primarily on OpenAlex metadata, including titles, abstracts, citation metadata, and associated identifiers, rather than systematic full-text analysis of all retrieved studies. Although DOI-linked text retrieval and accessible PDF parsing were performed when possible, methodological details appearing only within full manuscripts may not have been consistently captured.

Second, the retrieval and parsing procedures relied on rule-based and heuristic strategies using curated keyword libraries, regular-expression pattern sets, and model-family mappings. While these procedures enabled scalable comparative analysis across heterogeneous biomedical domains, they also introduce potential keyword bias, terminology sensitivity, and classification ambiguity. Studies using uncommon terminology, recently emerging model names, or highly domain-specific phrasing may have been under-detected, while ambiguous terminology may occasionally have produced false-positive matches.

Third, candidate studies were prioritized using combinations of dataset mentions, machine-learning keyword matches, regime-specific pattern matches, OpenAlex relevance scores, and citation counts. Although permissive filtering strategies and multiple dataset aliases were used to improve coverage, the retrieved corpora should not be interpreted as exhaustive representations of the broader biomedical machine-learning literature. Similarly, highly cited or more visible studies may have been preferentially retained relative to less cited recent work.

In addition, the analysis inherits known coverage biases associated with large scholarly metadata aggregators such as OpenAlex, including preferential coverage of indexed, English-language, and abstract-bearing publications.

Model extraction and dominant-model assignment were likewise performed using heuristic procedures. Exact model mentions were mapped to broader model families using curated pattern libraries, while dominant-model assignment relied on contextual cues derived from titles and abstracts. Although these procedures enabled consistent aggregation across large corpora, they cannot fully resolve ambiguity in hybrid or multi-stage pipelines where multiple models contribute substantially to the final methodology. Papers for which a unique dominant model could not be identified were excluded from the primary-only analyses.

Finally, the quantitative analyses presented in this work are intended primarily as descriptive computational literature-mapping statistics rather than formal bibliometric or inferential evaluations. The reported prevalence percentages and architectural-pattern frequencies should therefore be interpreted as approximate indicators of methodological tendencies within the retrieved corpora rather than definitive measurements of field-wide adoption frequencies. In addition, the rapidly evolving nature of multimodal, foundation-model, and deployment-oriented biomedical AI means that emerging architectures and terminology may continue to evolve beyond the temporal scope captured in the present review.

Despite these limitations, the proposed framework provides a scalable and reproducible mechanism for comparing methodological tendencies across heterogeneous biomedical datasets and prediction regimes. The computational workflow was intentionally designed to preserve intermediate auditability through retrieval logs, parsing summaries, model-family mappings, denominator tracking, and coverage statistics generated at multiple stages of the pipeline. These design choices were intended to support transparent comparative analysis while enabling future refinement and extension of the framework as biomedical machine-learning methodologies continue to evolve.

## 6. Conclusions

This review examined machine learning across heterogeneous biological, biomedical, behavioral, and environmental data representations, emphasizing that the central challenge is not simply combining heterogeneous data sources, but transforming them into predictive systems through representation, integration, and deployment-aware design.

Across structured, high-dimensional, multimodal, and temporal regimes, a consistent picture emerged. Different regimes favor different model families and reflect different assumptions about data structure, observability, modality composition and temporal organization. Classical statistical and machine learning methods remain important in structured and longitudinal settings, while deep representation learning has become central in high-dimensional and multimodal regimes. Rather than competing alternatives, these regimes can be viewed as complementary points along a continuum of expressivity and deployability.

The empirical patterns synthesized in this review are broadly consistent with tendencies previously observed within individual biomedical machine-learning subdomains, including the continued importance of classical statistical approaches in structured prediction settings, the prominence of deep learning in image-centric and representation-learning applications, and the methodological heterogeneity of multimodal systems. Rather than introducing entirely new empirical observations, the computational literature-mapping analyses were intended primarily to support comparative synthesis across heterogeneous datasets and prediction regimes within a unified biomedical machine-learning framework.

A central argument of this review is that biomedical machine learning is increasingly a systems design problem in which representation learning, fusion strategies, multistage pipelines, modality availability, robustness, and deployment constraints often shape performance as much as model choice itself. The architectural motif analysis synthesized in this review suggests that modular and staged pipeline structures remain recurrent organizational patterns across several biomedical machine-learning regimes, with learned representations and multistage predictive architectures appearing more consistently than explicitly robustness-aware or transfer-aware designs. These observations should be interpreted as exploratory descriptive tendencies within the retrieved corpora rather than definitive measurements of architectural prevalence across the broader biomedical machine-learning literature.

This systems perspective also highlights a recurring tension between expressive models and deployable systems. Rich multimodal models can improve prediction but often depend on assumptions about data availability and resources that do not hold in practice. Simpler or constrained systems may sacrifice some theoretical performance while providing greater robustness and scalability.

More broadly, the review argues that biomedical machine learning should be understood not only as a problem of predictive modeling, but also as a problem of alignment between representation, observability, robustness, and deployment conditions. In this sense, many of the central challenges arise less from isolated algorithmic limitations than from the difficulty of designing systems that remain reliable under heterogeneous, incomplete, and operationally constrained biomedical environments.

Taken together, the review suggests that progress in deployment-aware biomedical machine learning depends not only on improved algorithms, but also on principled approaches to representation, multimodal integration, modularity, robustness, and deployment under real-world constraints. The present review focused primarily on predictive machine-learning systems operating across heterogeneous biomedical representations rather than on explicit mechanistic scale-coupling models from systems biology or computational physiology. Future interaction between these complementary traditions may nevertheless become increasingly important for translational biomedical AI and data-driven physiological modeling. 

## Figures and Tables

**Table 1:** Principal biomedical data modalities organized by biological and behavioral representation level.

Level	Modality Class	Examples	Data Structure	Temporal Resolution	Accessibility
Biological (molecular)	Omics	Genomics, proteomics, metabolomics	Very high-dimensional, static	Static/episodic	Low
Biological (tissue)	Imaging	Histopathology, MRI, CT	High-dimensional spatial	Static/episodic	Low
Physiological signals	Biomarkers/biosignals	CRP, glucose, cortisol, ECG, EEG, blood pressure	Low–medium dimensional, structured or time-series	Sparse or continuous	Medium
Clinical abstractions	Health records (EHR)	Diagnoses, medications, lab panels, clinical notes	Mixed (tabular + text)	Irregular longitudinal	Medium
Phenotypic (individual)	Anthropometrics	BMI, waist circumference, body composition	Low-dimensional, structured	Static/sparse	High
Behavioral (self-reported)	Surveys	Lifestyle, stress, SES, mental health	Low-dimensional, structured	Episodic	High
Behavioral (passive)	Digital sensing	Activity, sleep, mobility, smartphone usage	Time-series, high-frequency	Continuous	High
Contextual/environmental	External data	Weather, air pollution, geolocation, built environment	Structured/spatio-temporal	Continuous/aggregated	High

**Table 2:** Representative datasets and their association with the prediction regimes considered in this review. Filled circles (•) denote dominant usage, while open circles (∘) indicate secondary applications. S = structured biomedical prediction; H = high-dimensional biomedical signals; M = multimodal learning; T = temporal and longitudinal modeling.

Dataset	Primary Modality	S	H	M	T
TCGA [14]	Omics		•	∘	
GTEx [15]	Omics		•	∘	
UK Biobank (imaging) [16]	Imaging + clinical	∘	•	∘	
ADNI [17]	Imaging + clinical		•	•	•
CAMELYON [18]	Histopathology		•		
MIMIC-III/IV [19,20]	Clinical + time series	•			•
eICU [21]	Clinical	•			•
MIDUS [22,23]	Behavioral + clinical	•		∘	•
HRS [24]	Survey + clinical	•			•
ELSA [25]	Survey + clinical	•			•
NHANES [26]	Clinical + survey	•		∘	∘
StudentLife [27]	Smartphone sensing			•	•
mPower [28]	Mobile health			•	•
RADAR-CNS [29]	Wearables + clinical			•	•
OpenAQ [30]	Environmental	∘			•
NOAA [31]	Environmental	∘			•

**Table 3:** Validation metrics computed from manually audited subsets across the four prediction regimes. $A_{\mathrm{dom}}$ and $A_{\mathrm{dom}}^{*}$ denote dominant-family accuracy across all audited papers and across non-ambiguous papers only, respectively. $R_{\mathrm{amb}}$ denotes the ambiguity rate. $P_{\mathrm{fam}}$, $R_{\mathrm{fam}}$, and $F1_{\mathrm{fam}}$ denote micro-averaged precision, recall, and F1 score for prevalence-oriented multi-label model-family extraction. $P_{\mathrm{PDF}}$ denotes the proportion of manually audited papers for which accessible PDF or full-text content was used during validation rather than title/abstract-only text sources.

Metric	S	H	M	T
$A_{\mathrm{dom}}$	82.0%	70.0%	70.0%	82.0%
$A_{\mathrm{dom}}^{*}$	97.6%	81.1%	85.4%	95.3%
$R_{\mathrm{amb}}$	16.0%	26.0%	18.0%	14.0%
$P_{\mathrm{fam}}$	0.856	0.663	0.860	0.821
$R_{\mathrm{fam}}$	0.928	0.984	0.895	0.951
$F1_{\mathrm{fam}}$	0.890	0.792	0.877	0.881
$P_{\mathrm{PDF}}$	56.0%	52.0%	78.0%	60.0%

**Table 4:** Sensitivity of prevalence-oriented model-family extraction performance to alternative taxonomy-collapse scenarios. Reported values summarize the observed range of $F1_{\mathrm{fam}}$ across the evaluated family-collapse perturbations relative to the baseline taxonomy.

Regime	Baseline $F1_{\mathrm{fam}}$	Collapse-Scenario Range	Maximum Absolute Change
S	0.890	0.889–0.900	0.011
H	0.792	0.797–0.845	0.053
M	0.877	0.879–0.913	0.036
T	0.881	0.879–0.889	0.010

**Table 5:** Structured data regime: distribution of dominant model families across datasets. Values represent the percentage of papers in which a single main modeling approach could be identified.

Model Family	eICU	ELSA	HRS	MIDUS	MIMIC	NHANES	NOAA	OpenAQ	UKBio	Total
Linear/GLM	34.8	81.5	78.0	85.5	23.6	86.1	52.6	20.0	48.5	53.6
Deep learning	7.9	4.4	0.0	1.6	19.1	0.0	5.3	25.0	25.0	11.9
Tree-based ensemble	29.3	2.2	0.0	3.2	36.3	3.8	5.3	40.0	8.7	15.8
Kernel/instance-based	0.0	0.7	0.0	0.0	3.2	0.0	0.0	0.0	2.0	1.1
Survival models	15.9	8.1	18.0	9.7	8.9	8.9	0.0	0.0	9.2	10.3
Probabilistic/statistical	1.2	0.0	4.0	0.0	0.0	0.0	0.0	0.0	2.0	0.9
Ensemble/stacking	6.1	2.2	0.0	0.0	5.1	0.0	31.6	15.0	3.1	4.1
Other classical ML	4.9	0.7	0.0	0.0	3.8	1.3	5.3	0.0	1.5	2.3
Papers (N)	164	135	50	62	157	79	19	20	196	882

**Table 6:** Structured data regime: prevalence of model families across datasets. Values represent the percentage of papers that use at least one model from the corresponding family; a single paper may include multiple model families.

Model Family	eICU	ELSA	HRS	MIDUS	MIMIC	NHANES	NOAA	OpenAQ	UKBio	Total
Linear/GLM	57.0	72.6	63.4	70.2	57.6	82.1	37.9	37.1	43.1	57.3
Deep learning	37.3	24.6	14.1	20.2	49.7	15.8	41.4	77.1	71.4	44.7
Tree-based ensemble	45.2	11.4	7.0	4.8	56.6	8.4	3.4	45.7	14.8	27.7
Kernel/instance-based	16.7	4.6	8.5	0.0	31.7	2.1	0.0	11.4	8.4	13.2
Survival models	26.2	22.9	26.8	15.5	18.3	18.9	0.0	0.0	16.2	19.2
Probabilistic/statistical	9.1	8.0	11.3	6.0	7.6	3.2	0.0	17.1	14.6	9.6
Ensemble/stacking	16.3	6.3	1.4	0.0	18.3	2.1	27.6	37.1	10.8	12.1
Other classical ML	20.5	6.3	0.0	0.0	25.9	2.1	3.4	14.3	5.9	12.0
Papers (N)	263	175	71	84	290	95	29	35	371	1413

**Table 7:** High-dimensional regime: distribution of dominant model families across datasets. Values represent the percentage of papers in which a single main modeling approach could be identified.

Model Family	ADNI	CAMELYON	GTEx	TCGA	UKBio	Total
Linear/GLM	27.5	0.0	61.1	39.0	50.5	35.5
Deep learning	28.9	84.7	8.3	8.0	23.3	29.9
Tree-based ensemble	10.1	2.0	3.7	8.0	6.8	6.5
Kernel/instance-based	18.8	1.0	0.9	5.0	1.9	6.6
Survival models	2.7	2.0	19.4	29.0	10.7	12.0
Probabilistic/statistical	1.3	0.0	2.8	0.0	1.0	1.1
Ensemble/stacking	8.7	3.1	2.8	3.0	5.8	5.0
Other classical ML	2.0	7.1	0.9	8.0	0.0	3.4
Papers (N)	149	98	108	100	103	558

**Table 8:** High-dimensional regime: prevalence of model families across datasets. Values represent the percentage of papers that use at least one model from the corresponding family; a single paper may include multiple model families.

Model Family	ADNI	CAMELYON	GTEx	TCGA	UKBio	Total
Linear/GLM	31.4	4.0	58.2	34.0	46.0	34.0
Deep learning	64.9	95.5	54.9	41.2	77.5	67.0
Tree-based ensemble	25.3	6.0	14.7	13.4	15.0	15.3
Kernel/instance-based	38.4	5.5	12.5	12.9	10.7	17.1
Survival models	9.0	1.5	39.7	69.6	15.0	25.8
Probabilistic/statistical	12.2	0.0	17.9	2.6	11.8	8.9
Ensemble/stacking	22.4	8.0	8.2	10.8	16.6	13.6
Other classical ML	11.4	7.5	2.2	10.8	5.9	7.8
Papers (N)	245	201	184	194	187	1011

**Table 9:** Multimodal regime: distribution of dominant model families across datasets. Values represent the percentage of papers in which a single main modeling approach could be identified.

Model Family	ADNI	GTEx	mPower	NHANES	RADAR-CNS	StudentLife	TCGA	UKBio	Total
Linear/GLM	22.6	53.8	27.8	0.0	15.2	18.6	25.9	54.5	29.1
Deep learning	24.5	7.7	16.7	0.0	18.2	32.9	11.1	25.5	22.5
Tree-based ensemble	15.1	0.0	29.6	0.0	12.1	20.0	14.8	10.9	17.0
Kernel/instance-based	13.2	0.0	1.9	0.0	9.1	2.9	7.4	0.0	4.9
Survival models	5.7	30.8	1.9	0.0	9.1	2.9	29.6	3.6	7.5
Probabilistic/statistical	1.9	0.0	5.6	0.0	15.2	2.9	0.0	0.0	3.6
Ensemble/stacking	15.1	7.7	9.3	0.0	15.2	4.3	7.4	3.6	8.5
Other classical ML	1.9	0.0	7.4	100.0	6.1	15.7	3.7	1.8	6.9
Papers (N)	53	13	54	1	33	70	27	55	306

**Table 10:** Multimodal regime: prevalence of model families across datasets. Values represent the percentage of papers that use at least one model from the corresponding family; a single paper may include multiple model families.

Model Family	ADNI	GTEx	mPower	NHANES	RADAR-CNS	StudentLife	TCGA	UKBio	Total
Linear/GLM	41.2	53.8	43.3	50.0	25.7	32.4	42.3	59.0	41.7
Deep learning	76.3	69.2	77.3	50.0	79.7	68.5	57.7	82.0	74.2
Tree-based ensemble	48.5	19.2	51.5	0.0	36.5	46.8	21.2	28.0	39.4
Kernel/instance-based	59.8	19.2	39.2	0.0	23.0	36.0	23.1	14.0	32.9
Survival models	12.4	38.5	5.2	50.0	8.1	6.3	53.8	10.0	14.1
Probabilistic/statistical	16.5	15.4	22.7	0.0	24.3	16.2	5.8	16.0	17.4
Ensemble/stacking	46.4	19.2	35.1	0.0	25.7	16.2	23.1	21.0	27.5
Other classical ML	20.6	0.0	25.8	50.0	12.2	31.5	15.4	13.0	19.9
Papers (N)	97	26	97	2	74	111	52	100	559

**Table 11:** Temporal regime: distribution of dominant model families across datasets. Values represent the percentage of papers in which a single main modeling approach could be identified. Abbreviations: AD = ADNI; eI = eICU; EL = ELSA; HR = HRS; MI = MIDUS; MM = MIMIC; mP = mPower; NH = NHANES; NO = NOAA; OA = OpenAQ; RA = RADAR-CNS; SL = StudentLife.

Model Family	AD	eI	EL	HR	MI	MM	mP	NH	NO	OA	RA	SL	Total
Linear/GLM	33.3	35.8	85.0	67.4	74.3	21.5	36.8	75.0	13.0	11.4	26.1	18.9	42.3
Deep learning	17.9	11.5	0.7	0.0	2.9	16.9	19.1	0.0	4.3	31.4	15.2	35.8	12.7
Tree-based ensemble	12.0	23.6	2.0	0.0	0.0	38.5	17.6	6.2	2.2	34.3	10.9	22.6	16.2
Kernel/instance-based	12.8	0.0	0.7	0.0	0.0	0.8	1.5	0.0	0.0	0.0	13.0	1.9	2.8
Survival models	9.4	17.6	8.2	25.6	20.0	14.6	4.4	15.6	0.0	0.0	6.5	1.9	10.9
Probabilistic/statistical	1.7	1.4	0.0	7.0	2.9	0.0	5.9	0.0	8.7	0.0	13.0	1.9	2.6
Ensemble/stacking	10.3	6.1	2.7	0.0	0.0	6.2	8.8	0.0	60.9	20.0	10.9	5.7	9.1
Other classical ML	2.6	4.1	0.7	0.0	0.0	1.5	5.9	3.1	10.9	2.9	4.3	11.3	3.4
Papers (N)	117	148	147	43	35	130	68	32	46	35	46	53	900

**Table 12:** Temporal regime: prevalence of model families across datasets. Values represent the percentage of papers that use at least one model from the corresponding family; a single paper may include multiple model families. Abbreviations: AD = ADNI; eI = eICU; EL = ELSA; HR = HRS; MI = MIDUS; MM = MIMIC; mP = mPower; NH = NHANES; NO = NOAA; OA = OpenAQ; RA = RADAR-CNS; SL = StudentLife.

Model Family	AD	eI	EL	HR	MI	MM	mP	NH	NO	OA	RA	SL	Total
Linear/GLM	43.6	60.6	75.5	57.6	52.6	61.7	44.3	70.0	15.8	29.0	27.4	37.9	52.0
Deep learning	55.2	37.6	18.6	16.9	28.1	54.2	68.7	32.5	24.6	67.7	76.4	73.6	47.1
Tree-based ensemble	33.7	42.5	10.6	8.5	3.5	54.6	41.7	15.0	3.5	48.4	35.8	49.4	33.8
Kernel/instance-based	41.9	16.4	4.3	5.1	0.0	30.8	31.3	5.0	0.0	11.3	20.8	39.1	20.8
Survival models	17.4	30.1	23.4	37.3	29.8	25.1	5.2	37.5	0.0	0.0	7.5	6.9	19.6
Probabilistic/statistical	17.4	8.8	8.5	11.9	8.8	8.8	20.9	2.5	12.3	12.9	22.6	23.0	13.0
Ensemble/stacking	29.7	15.9	6.4	1.7	0.0	22.0	30.4	5.0	59.6	35.5	19.8	20.7	20.2
Other classical ML	15.7	17.7	5.3	0.0	0.0	27.8	25.2	5.0	10.5	16.1	12.3	29.9	16.2
Papers (N)	172	226	188	59	57	227	115	40	57	62	106	87	1396

## Data Availability

The computational literature-mining framework, retrieval corpora, parsing outputs, and supplementary methodological resources used in this study are publicly available at: https://github.com/AAAlecu/biomedical-ml-literature-mapping (accessed on 24 May 2026).

## Supplementary Materials

The following supporting information can be downloaded at: https://www.mdpi.com/article/10.3390/bioengineering13060683/s1. Table S1: Dataset catalog, aliases, primary modalities, and supported prediction regimes. Table S2: Selected OpenAlex fields. Table S3: Components used to rank retrieved OpenAlex records during corpus construction. Table S4: Complete broad machine-learning keyword list used during OpenAlex corpus filtering. Table S5: Retrieval-stage terms for regime S. Table S6: Retrieval-stage terms for regime H. Table S7: Retrieval-stage terms for regime M. Table S8: Retrieval-stage terms for regime T. Table S9: Full parser-stage regex library for regime S. Table S10: Full parser-stage regex library for regime H. Table S11: Full parser-stage regex library for regime M. Table S12: Full parser-stage regex library for regime T. Table S13: Complete exact-model regex library and family mapping. Table S14: Dominant-model cue patterns. Table S15: Outcomes of the dominant-model assignment procedure. Table S16: Accessible-paper flag auto-detection order. Table S17: Method-like headings used to prioritize matching windows. Table S18: Regex library for Handcrafted feature pipelines. Table S19: Regex library for Learned representation pipelines. Table S20: Regex library for Fusion-centric architectures. Table S21: Regex library for Sequential predictive pipelines. Table S22: Regex library for Robust/transfer-aware pipelines. Table S23: Denominator definitions used across quantitative analyses.
