# Supplementary material for: Machine Learning Across Heterogeneous Biomedical Data: Representation, Integration, and Deployable Systems
Source: Bioengineering (Basel). 2026 Jun 12;13(6):683. doi: 10.3390/bioengineering13060683 (PMC13295449; doi:10.3390/bioengineering13060683)
Supplement: Supplementary file 1 [file bioengineering-13-00683-s001.zip › bioengineering-4329106-supplementary.pdf]

Supplementary Materials

Scope. This supplementary file documents the complete rule-based computational workflow used for the manuscript analyses. It includes the dataset catalog, machine-learning keyword library, regime term and regex libraries, exact-model extraction patterns, model-family mappings, dominant-model heuristics, pipeline-architecture pattern libraries, filtering logic, and exported reproducibility artifacts.

1. OpenAlex dataset retrieval and candidate corpus construction

This process constructs dataset-specific OpenAlex candidate corpora. It queries one selected dataset restricted to one prediction regime, using dataset aliases and local keyword filtering over reconstructed titles and abstracts. Records are deduplicated by OpenAlex identifier, ranked, and saved as text and CSV outputs. Table S1 summarizes the datasets, aliases, and supported prediction regimes used in the retrieval framework.

Table S1. Dataset catalog, aliases, primary modalities, and supported prediction regimes.

Key	Primary modality	Regimes	Aliases
TCGA	Omics	H, M	TCGA; The Cancer Genome Atlas
GTEX	Omics	H, M	GTEx; Genotype-Tissue Expression; Genotype Tissue Expression
UKB_IMAGING	Imaging + clinical	S, H, M	UK Biobank; UK Biobank imaging; UK Biobank imaging study
ADNI	Imaging + clinical	H, M, T	ADNI; Alzheimer's Disease Neuroimaging Initiative; Alzheimers Disease Neuroimaging Initiative
CAMELYON	Histopathology	H	CAMELYON; CAMELYON16; CAMELYON17
MIMIC	Clinical + time series	S, T	MIMIC-III; MIMIC III; MIMIC-IV; MIMIC IV; Medical Information Mart for Intensive Care
EICU	Clinical	S, T	eICU; eICU Collaborative Research Database; eICU-CRD
MIDUS	Behavioral + clinical	S, M, T	MIDUS; Midlife in the United States; Midlife in the United States study
HRS	Survey + clinical	S, T	Health and Retirement Study; HRS
ELSA	Survey + clinical	S, T	ELSA; English Longitudinal Study of Ageing; English Longitudinal Study of Aging
NHANES	Clinical + survey	S, M, T	NHANES; National Health and Nutrition Examination Survey
STUDENTLIFE	Smartphone sensing	M, T	StudentLife; StudentLife dataset
MPOWER	Mobile health	M, T	mPower; mPower dataset; mPower study; mPower Parkinson
RADAR_CNS	Wearables + clinical	M, T	RADAR-CNS; RADAR CNS; Remote Assessment of Disease and Relapse Central Nervous System
OPENAQ	Environmental	S, T	OpenAQ
NOAA	Environmental	S, T	NOAA; National Oceanic and Atmospheric Administration

1.1. OpenAlex API fields and ranking logic

For each dataset alias, the OpenAlex /works endpoint is queried using the search parameter. The selected OpenAlex fields are listed in Table S2.

Table S2. Selected OpenAlex fields.

Fields
Id
doi
title
display_name
publication_year
publication_date
type
cited_by_count
relevance_score
authorships
primary_location
abstract_inverted_index

Abstracts are reconstructed from the OpenAlex abstract_inverted_index field by mapping word positions back to ordered text. Records are deduplicated within each alias retrieval and again across aliases using the OpenAlex work identifier. The ranking components used during corpus construction are summarized in Table S3.

Table S3. Components used to rank retrieved OpenAlex records during corpus construction.

Ranking component	Implementation
Dataset mention	Boolean indicator based on whether any dataset alias appears in the combined title and abstract.
ML keyword match count	Number of broad machine-learning terms found in title and abstract.
Regime match count	Number of active regime terms found in title and abstract.
OpenAlex relevance score	OpenAlex relevance_score returned by the API.
Citation count	OpenAlex cited_by_count.

1.2. Broad machine-learning term library

A broad machine-learning keyword library was used during OpenAlex corpus filtering to identify potentially relevant studies, as shown in Table S4.

Table S4. Complete broad machine-learning keyword list used during OpenAlex corpus filtering.

Term	Term	Term
machine learning	artificial intelligence	deep learning
statistical learning	data mining	predictive modeling
prediction model	risk model	risk prediction
prediction	predictive	classifier
classification	regression model	multivariable model
multivariate model	algorithm	model development
logistic regression	linear regression	ridge regression
lasso	elastic net	generalized linear model
glm	cox	cox proportional hazards
survival model	survival analysis	time-to-event
time to event	hazard model	decision tree
classification tree	regression tree	random forest
extra trees	gradient boosting	gradient boosting machine
gbm	xgboost	lightgbm
catboost	adaboost	support vector machine
support vector machines	svm	neural network
neural networks	artificial neural network	ann
mlp	multilayer perceptron	deep neural network
dnn	convolutional neural network	cnn
recurrent neural network	rnn	lstm
transformer	attention model	naive bayes
bayesian model	bayesian network	hidden markov model
k-nearest neighbor	k nearest neighbor	knn
nearest neighbor	ensemble model	stacking
bagging	blending	auc
roc curve	area under the curve	model performance
cross-validation	cross validation	

1.3. Regime terms used during retrieval-stage filtering

Regime-specific retrieval terms were used to bias OpenAlex filtering toward structured (S), high-dimensional (H), multimodal (M), and temporal (T) prediction settings (Tables S5-S8).

Table S5. Retrieval-stage terms for regime S.

Term	Term	Term
tabular	structured data	structured
cohort	Survey	clinical variables
epidemiology	electronic health record	ehr
risk prediction	risk model	multivariable
population study	Biobank	

Table S6. Retrieval-stage terms for regime H.

Term	Term	Term
high-dimensional	high dimensional	omics
genomics	transcriptomics	proteomics
metabolomics	radiomics	histopathology
whole slide image	mri	fmri
ct	pet	image classification
segmentation	gene expression	microarray
sequencing		

Table S7. Retrieval-stage terms for regime M.

Term	Term	Term
multimodal	multi-modal	multimodality
data fusion	fusion model	sensor fusion
wearable	smartphone	mobile health
digital phenotyping	behavioral sensing	clinical and imaging
clinical + imaging	survey and clinical	

Table S8. Retrieval-stage terms for regime T.

Term	Term	Term
longitudinal	temporal	time series
timeseries	trajectory	trajectories
repeated measures	recurrent event	survival analysis
time-to-event	time to event	progression
forecasting	follow-up	follow up
incident		

2. Dataset-regime model extraction and model-family aggregation

The process involves parsing the OpenAlex CSV exports generated by the retrieval step. It applies regime-specific regex filtering, extracts exact model mentions using regex patterns, maps models into broader model families, assigns dominant models when possible, and exports both any-use and primary-only tables with audit logs.

2.1. Regime regex libraries used in paper-level relevance filtering

Curated regex libraries were used during parser-stage relevance filtering to identify papers associated with structured (S), high-dimensional (H), multimodal (M), and temporal (T) prediction regimes (Tables S9-S12).

Table S9. Full parser-stage regex library for regime S.

No.	Regex pattern
1	\btabular\b
2	\bstructured data\b
3	\bstructured clinical data\b
4	\bclinical variables?\b
5	\bdemographic[s]?\b
6	\bquestionnaire[s]?\b
7	\brisk factor[s]?\b
8	\bcovariate[s]?\b
9	\bmultivariable\b
10	\brisk prediction\b
11	\brisk model\b
12	\bclinical data\b
13	\belectronic health record[s]?\b
14	\behr\b
15	\bpopulation[-]based\b
16	\bepidemiolog(?:y|ical)\b
17	\bbiobank\b
18	\bcohort\b
19	\bsurvey\b

Table S10. Full parser-stage regex library for regime H.

No.	Regex pattern
1	\bhigh[-]dimensional\b
2	\bomics\b
3	\bgenomic[s]?\b
4	\btranscriptomic[s]?\b
5	\bproteomic[s]?\b
6	\bmetabolomic[s]?\b
7	\bgene expression\b
8	\bmicroarray\b
9	\bsequencing\b
10	\bnext[-]generation sequencing\b
11	\bradiomic[s]?\b
12	\bhistopatholog(?:y|ical)\b
13	\bwhole slide image[s]?\b
14	\bmri\b
15	\bfmri\b
16	\bct\b
17	\bpet\b
18	\bimage classification\b
19	\bsegmentation\b
20	\bvoxel\b
21	\bpixel[-]level\b

Table S11. Full parser-stage regex library for regime M.

No.	Regex pattern
1	\bmultimodal\b
2	\bmulti[-]modal\b
3	\bmultimodality\b
4	\bdata fusion\b
5	\bfusion model\b
6	\bfusion approach\b
7	\bmultisource\b
8	\bmulti[-]source\b
9	\bheterogeneous data\b
10	\bheterogeneous modalities\b
11	\bclinical and imaging\b
12	\bclinical \+ imaging\b
13	\bimaging and clinical\b
14	\behr and imaging\b
15	\behr and genomics\b
16	\bmulti[-]omics\b
17	\bwearable[s]?\b
18	\bsmartphone[s]?\b
19	\bmobile health\b
20	\bdigital phenotyping\b
21	\bbehavioral sensing\b
22	\bsensor fusion\b

Table S12. Full parser-stage regex library for regime T.

No.	Regex pattern
1	\blongitudinal\b
2	\btime[-]series\b
3	\btrajectory\b
4	\btrajectories\b
5	\bforecast(?:ing)?\b
6	\bfollow[-]up\b
7	\bover time\b
8	\btemporal\b
9	\bsequential\b
10	\bdynamic\b
11	\bincident\b
12	\bsurvival\b
13	\bhazard\b
14	\bprogression\b
15	\bnext \d+\s*(?:day|days|week|weeks|month|months|year|years)\b
16	\bwithin \d+\s*(?:h|hr|hrs|hour|hours|day|days|week|weeks|month|months|year|years)\b
17	\bprediction horizon\b
18	\bprospective\b
19	\bretrospective cohort\b

2.2. Regime relevance decision rule

For each paper, the parser computes a regime_score as the number of regime regex patterns matched in the combined text. For H, M, and T, a paper is considered relevant when the regime_score is greater than or equal to the configured min-regime-score. For S, permissive logic is used: a paper is retained if it has at least one structured cue, or if it contains identifiable ML models and has no stronger H, M, or T cues.

2.3. Exact-model extraction patterns and model-family mapping

Rule-based regex patterns were used to extract exact machine-learning model mentions and map them into broader model-family categories during corpus parsing (Table S13).

Table S13. Complete exact-model regex library and family mapping.

Exact model	Model family	Regex patterns
Autoencoder	Deep learning	\bauto[-]?encoder[s]?\b<br>\bvariational auto[-]?encoder[s]?\b<br>\bvae\b
CNN	Deep learning	\bcnn\b<br>convolutional neural network[s]?<br>\bconvnet[s]?\b
DNN	Deep learning	\bdnn\b<br>deep neural network[s]?<br>deep learning
GRU	Deep learning	\bgru\b<br>gated recurrent unit[s]?
Graph neural network	Deep learning	graph neural network[s]?<br>\bgnn\b<br>graph convolutional network[s]?<br>\bgcn\b
LSTM	Deep learning	\blstm\b<br>long short[-]term memory
MLP	Deep learning	\bmlp\b<br>multilayer perceptron<br>multi[-]layer perceptron
RNN	Deep learning	\brnn\b<br>recurrent neural network[s]?
TCN	Deep learning	temporal convolutional network[s]?<br>\btcn\b
Transformer	Deep learning	\btransformer[s]?\b<br>bert\b<br>attention[-]based
AdaBoost	Tree-based ensemble	\badaboost\b
CatBoost	Tree-based ensemble	\bcatboost\b

Extra Trees	Tree-based ensemble	extra trees<br>extremely randomized trees
Gradient Boosting	Tree-based ensemble	gradient boosting<br>gradient boosted trees?
LightGBM	Tree-based ensemble	\blightgbm\b
Random Forest	Tree-based ensemble	random forest[s]?<br>\brf\b
XGBoost	Tree-based ensemble	\bxgboost\b<br>extreme gradient boosting
Elastic Net	Linear / GLM	elastic net
LASSO	Linear / GLM	\blasso\b
Linear Regression	Linear / GLM	linear regression
Logistic Regression	Linear / GLM	logistic regression<br>\blogit\b
Ridge Regression	Linear / GLM	ridge regression
Support Vector Machine	Kernel / instance-based	support vector machine[s]?<br>\bsvm\b<br>support vector regression<br>\bsvr\b
k-Nearest Neighbors	Kernel / instance-based	k[-]nearest neighbors?<br>\bknn\b
Cox Proportional Hazards	Survival models	cox proportional hazards?<br>cox regression<br>\bcox model
Joint Model	Survival models	joint model[s]?
Kaplan-Meier	Survival models	kaplan[--]meier
Random Survival Forest	Survival models	random survival forest[s]?
ARIMA	Probabilistic / statistical	\barima\b
Bayesian Regression	Probabilistic / statistical	bayesian regression<br>bayes(?:ian)? model[s]?
Generalized Estimating Equations	Probabilistic / statistical	generalized estimating equations?<br>\bgee\b
Hidden Markov Model	Probabilistic / statistical	hidden markov model[s]?<br>\bhmm\b
Kalman Filter	Probabilistic / statistical	kalman filter[s]?
Markov Model	Probabilistic / statistical	markov model[s]?
Mixed-Effects Model	Probabilistic / statistical	mixed[-]effects? model[s]?<br>mixed model[s]?<br>multilevel model[s]?
Naive Bayes	Probabilistic / statistical	naive bayes
Ensemble	Ensemble / stacking	\bensemble\b<br>stacking<br>stacked ensemble<br>blending
Decision Tree	Other classical ML	decision tree[s]?
Federated Learning	Other classical ML	federated learning
Reinforcement Learning	Other classical ML	reinforcement learning

2.4. Dominant-model assignment heuristics

The parser assigns a single primary model only when a unique highest-scoring model mention can be identified. Each detected exact model receives contextual cue points and a base mention point. If there is a tie among highest-scoring models, no dominant model is assigned and the paper is marked primary_unclear.

Heuristic contextual scoring was used to assign dominant models during primary-only analysis (Table S14), while the resulting assignment outcomes and exclusion categories are summarized in Table S15.

Table S14. Dominant-model cue patterns.

No.	Cue regex template	Score contribution
1	we (?:developed|proposed|used|trained|applied|implemented|constructed) (?:an?|the)?\s*{name}	+2 if matched
2	{name} (?:model|classifier|regressor|framework|approach)	+2 if matched
3	using (?:an?|the)?\s*{name}	+2 if matched
4	based on (?:an?|the)?\s*{name}	+2 if matched
Base mention	Escaped exact model name appears anywhere in text	+1

Table S15. Outcomes of the dominant-model assignment procedure.

Primary assignment outcome	Meaning
ok	A unique highest-scoring model was identified and assigned as the primary model.
no_identifiable_model	No exact model was detected.
primary_unclear	No unique winner was available after scoring, usually because of ties or ambiguity.
not_included_any	The paper was not included in any-use analysis because it was not both relevant and model-identifiable.

3. Pipeline-architecture pattern extraction from audit logs

This process operates on already audited paper corpora rather than querying OpenAlex again. It uses model-extraction audit logs as the denominator backbone, optionally recovers metadata from the original OpenAlex CSV files, optionally retrieves DOI/PDF text, extracts pipeline architecture motifs, and exports prevalence, count, coverage, and evidence tables.

Pipeline-architecture pattern extraction was performed on previously audited paper corpora using audit-log inclusion flags and method-oriented text windows to prioritize architectural pattern matching (Tables S16-S17).

Table S16. Accessible-paper flag auto-detection order.

Priority	Audit-log flag
1	include_any_use
2	used_any
3	include_primary_only
4	used_primary

Table S17. Method-like headings used to prioritize matching windows.

Heading	Heading	Heading
methods	materials and methods	methodology
model	models	model architecture
architecture	approach	framework
pipeline	experiments	implementation
statistical analysis	machine learning	

3.1. Complete pipeline-architecture pattern taxonomy

The pipeline-architecture analysis framework classified higher-level methodological workflows into five major architectural categories: handcrafted feature pipelines, learned representation pipelines, fusion-centric architectures, sequential predictive pipelines, and robust or transfer-aware pipelines. These categories were identified using curated regex libraries applied to method-oriented text windows extracted from titles, abstracts, DOI landing pages, and accessible PDF content.

3.1.1. Handcrafted feature pipelines

Handcrafted feature pipelines were identified using regex patterns associated with manual feature engineering, radiomics, engineered biomarkers, and classical handcrafted descriptors (Table S18).

Table S18. Regex library for Handcrafted feature pipelines.

No.	Regex pattern
1	\bfeature engineering\b
2	\bhandcrafted features?\b
3	\bhand[-]crafted features?\b
4	\bengineered features?\b
5	\bmanual feature extraction\b
6	\bfeature selection\b
7	\bfeature screening\b
8	\bfeature ranking\b
9	\bfeature construction\b
10	\bfeature design\b
11	\bradiomic features?\b
12	\bradiomics\b
13	\btexture features?\b
14	\bmorphological features?\b
15	\bshape descriptors?\b
16	\bwavelet features?\b
17	\bspectral features?\b
18	\bfrequency-domain features?\b
19	\btime-domain features?\b
20	\bbiomarker panel\b
21	\bengineered predictors?\b

3.1.2. Learned representation pipelines

Learned representation pipelines were identified using regex patterns related to representation learning, embeddings, autoencoders, latent spaces, self-supervised learning, and pretrained encoders (Table S19).

Table S19. Regex library for Learned representation pipelines.

No.	Regex pattern
1	\brepresentation learning\b
2	\blearned representation(s)?\b
3	\blearned feature representation(s)?\b
4	\blearned latent representation(s)?\b
5	\blatent representation(s)?\b
6	\blatent space\b
7	\blatent embedding(s)?\b
8	\blatent feature(s)?\b
9	\bembedding learning\b
10	\blearned embedding(s)?\b
11	\bfeature embedding(s)?\b
12	\bpatient embedding(s)?\b
13	\bjoint embedding(s)?\b
14	\bshared embedding(s)?\b
15	\bautoencoder(s)?\b
16	\bauto-encoder(s)?\b
17	\bvariational autoencoder(s)?\b
18	\bvariational auto-encoder(s)?\b
19	\bVAE\b
20	\bdenoising autoencoder(s)?\b
21	\bsparse autoencoder(s)?\b
22	\bself-supervised learning\b
23	\bcontrastive learning\b
24	\bcontrastive embedding(s)?\b
25	\bdeep feature learning\b
26	\bdeep feature extraction\b
27	\bdeep feature(s)?\b
28	\bfeature learning\b
29	\bpretrained representation\b
30	\bpre-trained representation\b
31	\bpretrained encoder\b
32	\bpre-trained encoder\b
33	\bfoundation model\b
34	\bencoder-decoder\b

35	\bencoder decoder\b
36	\btransformer encoder\b
37	\bgraph embedding(s)?\b
38	\bnode embedding(s)?\b
39	\bsequence embedding(s)?\b
40	\bimage embedding(s)?\b
41	\btext embedding(s)?\b
42	\bdimensionality reduction\b
43	\bmanifold learning\b

3.1.3. Fusion-centric architectures

Fusion-centric architectures were identified using regex patterns associated with multimodal fusion, cross-modal attention, multimodal transformers, and multi-view integration strategies (Table S20).

Table S20. Regex library for Fusion-centric architectures.

No.	Regex pattern
1	\bmultimodal fusion\b
2	\bmulti[-]modal fusion\b
3	\bfusion model\b
4	\bfusion architecture\b
5	\bfusion framework\b
6	\bfusion network\b
7	\bearly fusion\b
8	\blate fusion\b
9	\bintermediate fusion\b
10	\bhybrid fusion\b
11	\bfeature fusion\b
12	\bdecision fusion\b
13	\bscore fusion\b
14	\btensor fusion\b
15	\bmultimodal transformer\b
16	\bcross[-]modal attention\b
17	\bcross modality attention\b
18	\bcross[-]modal\b
19	\bco[-]attention\b
20	\battention fusion\b
21	\bjoint representation learning\b
22	\bmultimodal integration\b
23	\bmulti[-]modal integration\b
24	\bmultiview learning\b
25	\bmulti[-]view learning\b
26	\bmulti[-]view fusion\b

3.1.4. Sequential predictive pipelines

Sequential predictive pipelines were identified using regex patterns associated with multi-stage prediction workflows, cascaded architectures, stacked ensembles, hierarchical pipelines, and sequential risk propagation strategies (Table S21).

Table S21. Regex library for Sequential predictive pipelines.

No.	Regex pattern
1	\btwo-stage model\b
2	\btwo stage model\b
3	\btwo-stage prediction\b
4	\btwo step model\b
5	\bmulti-stage model\b
6	\bmultistage model\b
7	\bmulti-stage prediction\b
8	\bmulti-step prediction\b
9	\bstacked model\b
10	\bstacked classifier\b
11	\bstacked generalization\b
12	\bstacked ensemble\b
13	\bstacking ensemble\b

14	\bcascade(d)? model\b
15	\bcascade(d)? prediction\b
16	\bcascaded pipeline\b
17	\bhierarchical model\b
18	\bhierarchical prediction\b
19	\bhierarchical pipeline\b
20	\bcoarse-to-fine\b
21	\bcoarse to fine\b
22	\bsequential prediction\b
23	\bprogressive prediction\b
24	\bprogressive learning\b
25	\bstage[-]1\b.*\bstage[-]2\b
26	\bfirst[-]stage model\b
27	\bsecond[-]stage model\b
28	\bprediction.*fed into\b
29	\bpredicted .* used as (?:an)?input\b
30	\boutput of .* used as (?:an)?input\b
31	\brisk score.*used as (?:an)?input\b
32	\bmeta-classifier\b
33	\bmeta classifier\b
34	\bmeta-learner\b

3.1.5. Robust / transfer-aware pipelines

Robust and transfer-aware pipelines were identified using regex patterns associated with knowledge distillation, teacher–student learning, modality robustness, model compression, and graceful degradation under missing modalities (Table S22).

Table S22. Regex library for Robust / transfer-aware pipelines.

No.	Regex pattern
1	\bknowledge distillation\b
2	\bdistillation\b
3	\bteacher-student\b
4	\bteacher student\b
5	\bteacher model\b.*\bstudent model\b
6	\bstudent network\b
7	\bdistilled model\b
8	\bmodel compression\b
9	\bmodel pruning\b
10	\bquantization aware\b
11	\bmissing modality\b
12	\bmissing modalities\b
13	\bmissing-modality\b
14	\bpartial modality\b
15	\bincomplete modality\b
16	\bmodality dropout\b
17	\bmodality imputation\b
18	\bmodality invariant\b
19	\bmodality-invariant\b
20	\brobust multimodal\b
21	\brobust fusion\b
22	\bgraceful degradation\b

4. Interpretation of count denominators and percentage tables

Different quantitative analyses used different denominator definitions depending on the filtering and inclusion criteria applied at each stage of the computational workflow. The denominator definitions and interpretation of percentages used throughout the supplementary analyses are summarized in Table S23.

Table S23. Denominator definitions used across quantitative analyses.

Analysis	Denominator	Interpretation
Any-use model-family table	Papers that passed regime relevance filtering and contained at least one identifiable ML model.	A paper may contribute to multiple exact models and multiple families; percentages need not sum to 100%.
Primary-only model-family table	Papers for which a unique dominant model could be identified.	Each paper contributes to exactly one family; ambiguous papers are excluded.
Pipeline-pattern prevalence table	Accessible/analyzable papers from the audit log with enough text to assess.	A paper may contain multiple pipeline motifs; percentages need not sum to 100%.
Coverage table	Accessible papers from the audit log.	Tracks assessable text availability and whether at least one pipeline motif was detected.

5. Manual audit validation and taxonomy sensitivity analysis

To evaluate the reliability of the computational literature-mining framework, an additional manual audit procedure was performed across the four prediction regimes (structured, high-dimensional, multimodal, and temporal). For each regime, a random audit subset of 50 papers was sampled from the intermediate audit logs after relevance filtering. Eligible papers were sampled from studies retained within the final regime-specific analysis corpora after computational relevance filtering. The audit therefore evaluated the reliability of the downstream model-extraction and dominant-family assignment procedures on the papers contributing to the quantitative summaries reported throughout the manuscript. Accessible PDF files were downloaded when available; otherwise, title and abstract text reconstructed from OpenAlex metadata were used for manual review.

Manual annotations included: (i) dominant model family, (ii) dominant exact model, (iii) ambiguity flags for papers without a uniquely identifiable dominant model, and (iv) all identifiable exact models and model families present within the paper. Validation metrics were computed separately for dominant-model-family assignment and prevalence-oriented model-family extraction. Prevalence-oriented extraction metrics were computed using micro-averaged multi-label precision, recall, and F1 across all manually annotated family assignments within each regime-specific audit subset.

Additional robustness analyses evaluated sensitivity to alternative coarser model-family taxonomies by simultaneously collapsing automated and manual family labels into broader aggregate categories. Evaluated perturbations included collapsing multiple classical machine-learning families into broader aggregate groups, merging ensemble-oriented families, merging survival and linear/statistical families, and collapsing all non-deep-learning families into a single category. These analyses were designed to evaluate whether the broad methodological conclusions depended strongly on fine-grained model-family definitions.

The complete source code, regex libraries, taxonomy definitions, audit CSVs, validation scripts, and sensitivity-analysis outputs are publicly available in the accompanying GitHub repository.
